# Enhanced antidepressant effects of BDNF-quercetin alginate nanogels for depression therapy

**DOI:** 10.1186/s12951-023-02150-4

**Published:** 2023-10-18

**Authors:** Dong Xu, Li-Na Gao, Xu-Jiao Song, Qin-Wei Dong, Yi-Bing Chen, Yuan-Lu Cui, Qiangsong Wang

**Affiliations:** 1 State Key Laboratory of Component-based Chinese Medicine, Research Center of Traditional Chinese Medicine, Tianjin University of Traditional Chinese Medicine, Tianjin, 301617 China; 2 Haihe Laboratory of Modern Chinese Medicine, Tianjin, 301617 China; 3 State Key Laboratory of Advanced Medical Materials and Devices, Engineering Research Center of Pulmonary and Critical Care Medicine Technology and Device (Ministry of Education), Tianjin Key Laboratory of Biomedical Materials, Institute of Biomedical Engineering, Chinese Academy of Medical Science & Peking Union Medical College, Tianjin, 300192 China; 4 Shandong Collaborative Innovation Center for Diagnosis, Treatment and Behavioral Interventions of Mental Disorders, Institute of Mental Health, College of Pharmacy, Jining Medical University, Jining, Shandong 272067 China

**Keywords:** Quercetin, BDNF, Depressive disorder, Intranasal delivery, Nanogels

## Abstract

**Background:**

Brain-derived neurotrophic factor (BDNF) with neuronic development and function is a promising therapeutic agent for treating depressive disorder, according to the neurotrophin hypothesis. However, the delivery of BDNF into the brain is not easy as these large protein molecules cannot efficiently cross the blood-brain barrier (BBB) and easily suffer oxidative damage in vivo. Therefore, the quercetin-based alginate nanogels (quercetin nanogels) loaded with BDNF have been developed, which could efficiently bypass the BBB via the nose-to-brain pathway and protect BDNF from oxidative damage, providing an effective route for the therapy of depressive disorders by intranasal delivery.

**Results:**

Quercetin nanogels exhibited uniform size distribution, excellent biocompatibility, and potent antioxidant and anti-inflammatory activities. Quercetin nanogels in the thermosensitive gel achieved sustained and controlled release of BDNF with non-Fick’s diffusion, exhibited rapid brain distribution, and achieved nearly 50-fold enhanced bioavailability compared to oral quercetin. Quercetin nanogels as a therapeutic drug delivery carrier exerted antidepressant effects on reserpine-induced rats, effectively delivered BDNF to reverse despair behavior in stress-induced mice, and exhibited antidepressant effects on chronic mild unpredictable stimulation (CUMS) rats. These antidepressant effects of BDNF-Quercetin nanogels for CUMS rats are associated with the regulation of the glutamatergic system, PI3K-Akt, and BDNF-TrkB signaling pathway.

**Conclusions:**

In this study, we provide a promising strategy for brain delivery of BDNF for treating depressive disorders, effectively achieved through combining quercetin nanogels and intranasal administration.

**Supplementary Information:**

The online version contains supplementary material available at 10.1186/s12951-023-02150-4.

## Highlights

Natural antioxidant quercetin-based alginate nanogels protect BDNF from oxidative damage and have a quick brain distribution.

Intranasal quercetin nanogels exhibit significantly higher bioavailability compared to oral quercetin.

BDNF-Quercetin nanogels in thermosensitive gel release BDNF and quercetin in a sustained and controlled manner.

BDNF-Quercetin nanogels in thermosensitive gel have significant antidepressant effects on stress-induced mice and rats.

**Supplementary Information:**

The online version contains supplementary material available at 10.1186/s12951-023-02150-4.

## Introduction

Depressive disorder is a globally prevalent mental health condition affecting over 264 million individuals. It is characterized by persistent sadness and a marked loss of interest or pleasure in previously rewarding and enjoyable activities [1]. According to the inflammatory theory of depression, compared to healthy individuals, depressed ones have a higher level of inflammation and an association with chronic inflammation. Individuals with depression have some inflammatory symptoms, such as peripheral and central inflammation, which are produced by altering brain-derived neurotrophic factor-tyrosine kinase receptor b (BDNF-TrkB) pathway in the brain region such as the prefrontal cortex, hippocampus, and nucleus accumbens [2]. BDNF, a crucial biomarker for the pathogenesis of depression, is also a valuable measure of the difference between healthy and depressed individuals. Reduced and elevated BDNF concentrations are linked to synaptic plasticity, neuronal atrophy, and survival and neuronal differentiation, respectively [3]. The deprivation of BDNF also increases inflammatory levels and susceptibility to behavioral and cognitive consequences in stress-induced mice [4]. Hence, exogenously supplying BDNF is a promising strategy for treating depressive disorder. Given the impracticality of intraventricular injections of BDNF for clinical application, extensive research is being conducted on local BDNF delivery via hydrogel systems or nanoscale carriers [5–7]. Delivering BDNF to the brain seems to be a hopeful treatment, but it poses challenges due to the limited ability of large protein molecules to traverse the BBB effectively and is easy to suffer in vivo oxidative damage. Hence, a better delivery strategy is essential.

All the time, due to the restriction of BBB on the delivery of therapeutics into the brain, intranasal delivery, as a noninvasive strategy of drug delivery, had become a potential therapy for brain diseases, such as protein delivery [8]. Intranasal delivery of neurotrophic factors has great clinical potential, such as simplicity, noninvasive, rapid, etc. [9, 10]. Intranasal drug administration can deliver drugs directly to the brain via the nose-to-brain pathway, further diffusing into other brain regions from beginning points of brain entry and attaining better therapeutic effects [11]. In 2019, the Food and Drug Administration (FDA) approved esketamine, a breakthrough intranasal antidepressant characterized by rapid relief of depressive symptoms. Esketamine represents a new therapeutic approach for people suffering from depression that does not respond to conventional treatments [12–14]. Based on this fact, BDNF was intranasally delivered to the brain, further would attain significant antidepressant effects.

Quercetin, as a natural antioxidant with antidepressant effects, is used to protect BDNF from in vivo oxidative damage and increases the antidepressant activities of BDNF. However, quercetin has poor water solubility, low bioavailability, poor permeability, and stability, which presents a limitation for biomedical applications [15]. Nanotechnology-based drug delivery systems contribute to improving poor solubility, rapid degradation, and transient biological activities but also selectively accumulate by targeting tissues, further reducing side effects [16, 17]. As reported in our previous studies, we have designed a “material-drug” structural nanodrug delivery system constructed by alginate and quercetin. The low solubility and bioavailability of quercetin, as a crosslinker and therapeutic drug, is improved by quercetin-alginate nanogels that present an antioxidant and protective effect in vitro and a remarkable ability to reverse the damage caused by oxidative stress in acute lung injury (ALI) rats [18]. Therefore, quercetin-alginate nanocarriers would be a promising choice to overcome the poor solubility of quercetin and deliver BDNF protein.

Although intranasal delivery had significant advantages like ease of administration, improved bioavailability, avoidance of first-pass metabolism, rapid-onset, and high patient compliance, the drug had a short residence time in the nasal cavity [19]. Environmentally responsive hydrogel is a good idea for drug delivery, such as thermosensitive network hydrogel [20]. In particular, thermosensitive hydrogels prepared by poloxamers showed a controlled in situ release for therapeutics [21], which is suitable for intranasal delivery.

**Fig. 1:**
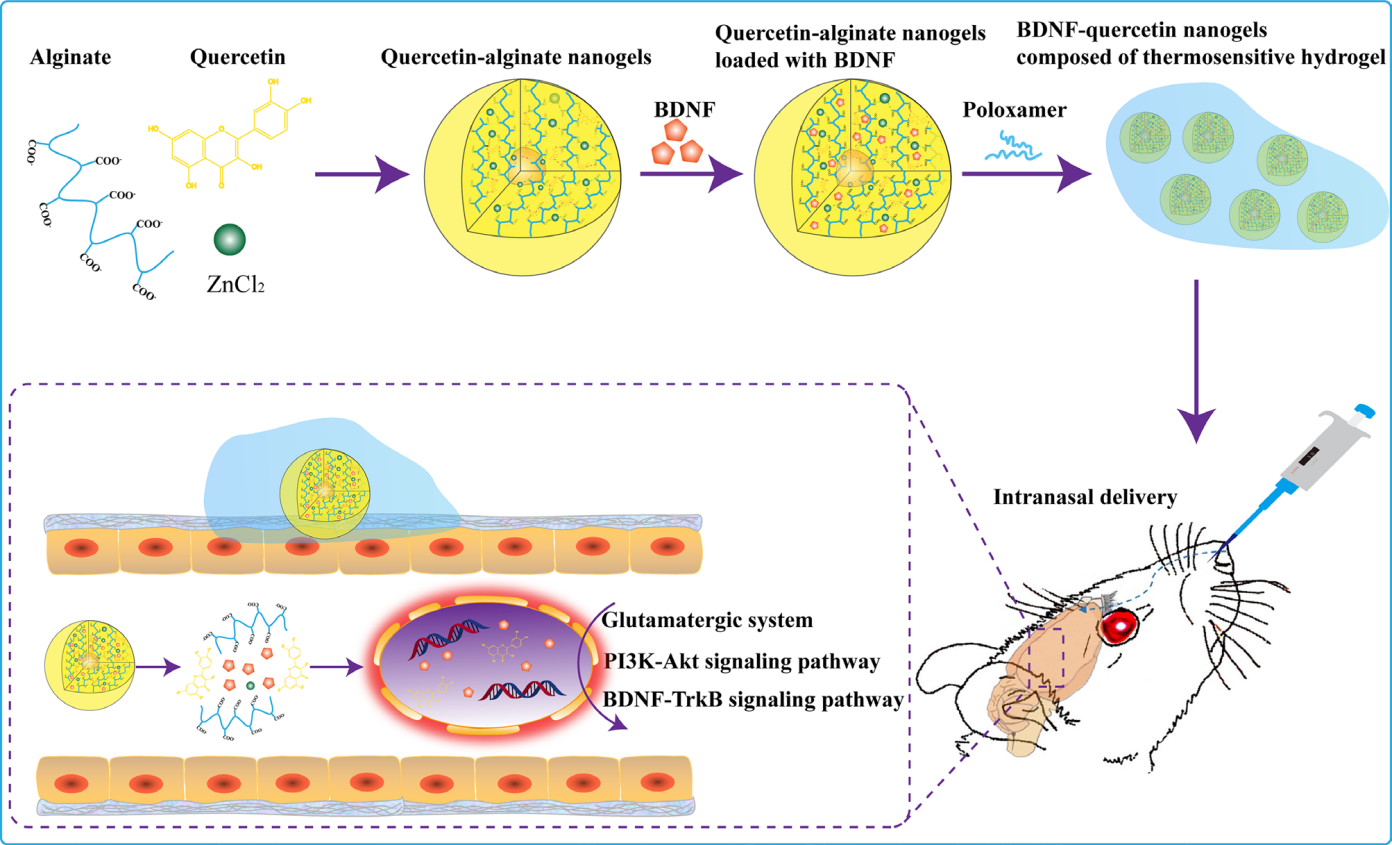
Design strategy and antidepressant mechanism of BDNF-Quercetin nanogels in the thermosensitive gel

Herein, we construct the quercetin-based alginate nanogels (quercetin nanogels) loaded with BDNF (BDNF-quercetin nanogels) composed of thermosensitive gel for the combination therapy of depression by intranasal delivery (Fig. 1**)**. BDNF-quercetin nanogels in thermosensitive gel present more prospects: (i) Natural antioxidant quercetin protects BDNF from oxidative damage. (ii) Quercetin nanogels attain a quick brain distribution and help to achieve the release of BDNF in a sustained and controlled manner. (iii) Increasing antidepressant effects on stress-induced mice and rats. Moreover, the antidepressant mechanisms of BDNF-quercetin nanogels on chronic mild unpredictable stimulation (CUMS) are investigated.

## Results and discussions

### Characterizations of quercetin nanogels and BDNF-Quercetin nanogels

According to the property that divalent cations bridge alginate by chain-chain association, the forming junction zones can encapsulate the poorly soluble drug [22]. Quercetin has vigorous antioxidant activities, but its solubility is poor, resulting in low bioavailability. Therefore, many nano-preparations, such as nanofibers [23], nanogels [24], and nanovesicles [25], were used to improve their poor bioavailability. To solve the same problem, quercetin nanogels as antioxidant carriers were prepared using zinc alginate based on a phase inversion emulsification method.

**Fig. 2:**
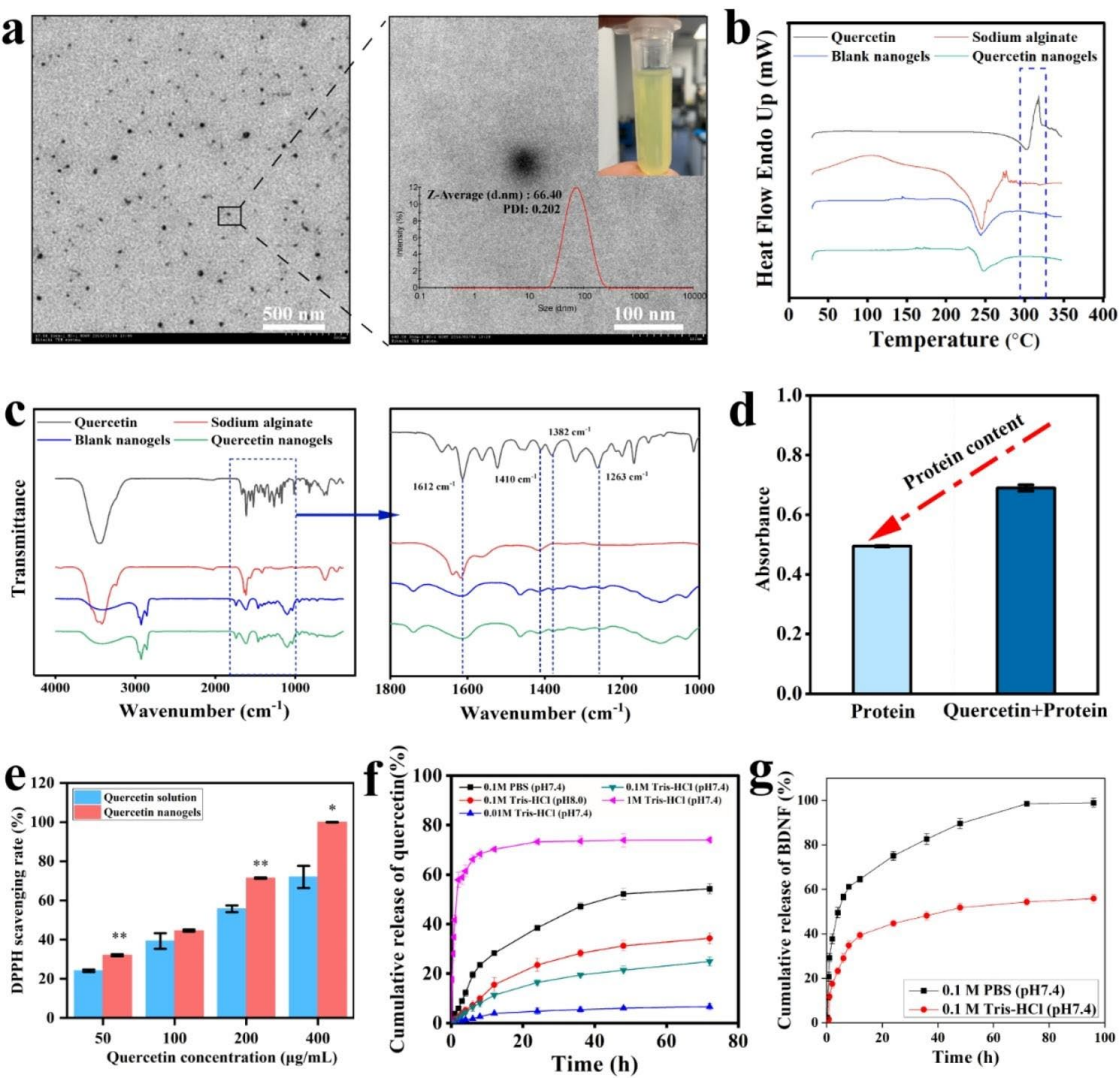
Characterization of quercetin nanogels and BDNF-Quercetin nanogels. **(a)** TEM image of quercetin nanogels (Left). Scale bar, 500 nm. The magnified TEM image of quercetin nanogels (Right). Scale bar, 100 nm. **(b)** The differential scanning calorimetry (DSC) patterns of quercetin nanogels. **(c)** The Fourier-transform infrared spectroscopy (FT-IR) of quercetin nanogels. **(d)** The protective effect of quercetin on H_2_O_2_-induced protein. **(e)** Antioxidant activities of quercetin nanogels. **(f)** Cumulative release profiles of quercetin nanogels. **(g)** Cumulative release profiles of BDNF-Quercetin nanogels

The morphology and particle size of quercetin nanogels were observed using transmission electron microscopy (TEM) and phase-analysis light scattering (PALS). The well-formed spherical quercetin nanogels (Fig. 2a), with a particle size of 76.34 ± 2.34 nm and zeta potentials of -14.48 ± 0.90 mV, presented transparent and yellow.

Differential scanning calorimetry (DSC) profiles provide valuable insights into the physical condition of the incorporated drug within the blend and the composite structure [26]. As depicted in Fig. 2b, quercetin exhibited an endothermic peak concurrent with a melting point at approximately 318 ºC, followed by an exothermic peak. Contrarily, sodium alginate and quercetin nanogels showed no peaks within the same temperature range. The absence of corresponding peaks in the quercetin nanogels suggested that quercetin was successfully encapsulated into quercetin nanogels, as inferred from the comparative analysis of the DSC profiles of the three components. Fourier-transform infrared spectroscopy (FT-IR) was shown in Fig. 2c. The band of quercetin-OH stretching appeared at 3300–3500 cm^− 1^. Quercetin had three sharp peaks, 1410^− 1^ cm, 1382^− 1^ cm, and 1263^− 1^ cm. Meanwhile, a similar peak of quercetin was presented at the quercetin nanogels, showing that quercetin was encapsulated into nanogels. By analyzing the characterization of particle size, TEM, DSC, FT-IR, Confocal micro-Raman spectroscopy (FigureS1**a**), and X-ray diffraction analysis (**Figure **S1**b**), the results demonstrated that quercetin nanogels, as a nanocarrier, were successfully prepared and quercetin was constructed into alginate nanogels using the method of phase inversion emulsification. Next, the loading capacities of quercetin and BDNF were determined using high-performance liquid chromatography and BDNF Emax immunoassay system, respectively. These results demonstrated the successful encapsulation of quercetin and BDNF within the nanogels, with loading capacities of 1.1 ± 0.02% and 0.018 ± 0.001%, respectively.

It was reported that oxidative stress suppressed and damaged the level of trophic factors such as BDNF [27]. Meanwhile, quercetin and its complexes with antioxidant activities had been widely applied for medicinal applications [28]. Therefore, as an antioxidant, quercetin protects BDNF delivery for brain delivery. It was indicated in Fig. 2d that protein induced by H_2_O_2_ had oxidative damage and a low absorbance when compared to protein combined with quercetin. Quercetin nanogels, compared to quercetin solution, also significantly improve antioxidant capacity with increasing concentration (Fig. 2e). These in vitro results suggested that quercetin, particularly quercetin nanogels, can be an antioxidant protein carrier that effectively prevents the protein from oxidative damage.

To verify whether quercetin was involved in constructing quercetin nanogels, the release behavior of quercetin nanogels was investigated using release media. The cumulative release rate of quercetin was 24.87 ± 1.87% in 0.1 M Tris-HCl (pH 7.4), 54.22 ± 2.02% in 0.1 M phosphate-buffered saline (PBS, pH 7.4), 34.21 ± 2.32% in 0.1 M Tris-HCl (pH 8.0), 6.67 ± 1.32% in 0.01 M Tris-HCl (pH 7.4), and 74.03 ± 1.32% in 1 M Tris-HCl (pH 7.4) (Fig. 2f). The cumulative release rates were modulated by varying the concentration and pH of Tris-HCl, which altered the release media’s osmotic pressure and ionic concentration. These results suggested that quercetin was not entirely released from quercetin nanogels. By collecting the remaining quercetin nanogels in the 0.1 M PBS (pH 7.4) and lysing them with sodium citrate, the quercetin content was found to be approximately 30%. The results showed that part of quercetin participated in the system construction of quercetin nanogels, strengthening the cross-linking between molecular chains and making the quercetin nanogels resistant to dissolution. The release behavior of BDNF-Quercetin nanogels is shown in Fig. 2g. After 12 h, the cumulative release rates of BDNF in 0.1 M PBS (pH 7.4) and 0.1 M Tris-HCl (pH 7.4) were 64.58 ± 1.29% and 39.40 ± 1.65%, respectively, which is because BDNF is more water-soluble and can freely diffuse into the release media. Finally, the cumulative release rate of BDNF reached 98.93 ± 1.98% in 0.1 M PBS (pH 7.4) and 55.87 ± 1.99% in 0.1 M Tris-HCl (pH 7.4), respectively, showing that monovalent cations of the release medium facilitate the release of BDNF. Overall, quercetin nanogels mainly release BDNF through free diffusion and ion exchange in the release medium.

### Characterization of BDNF-Quercetin nanogels in the thermosensitive gel

**Fig. 3:**
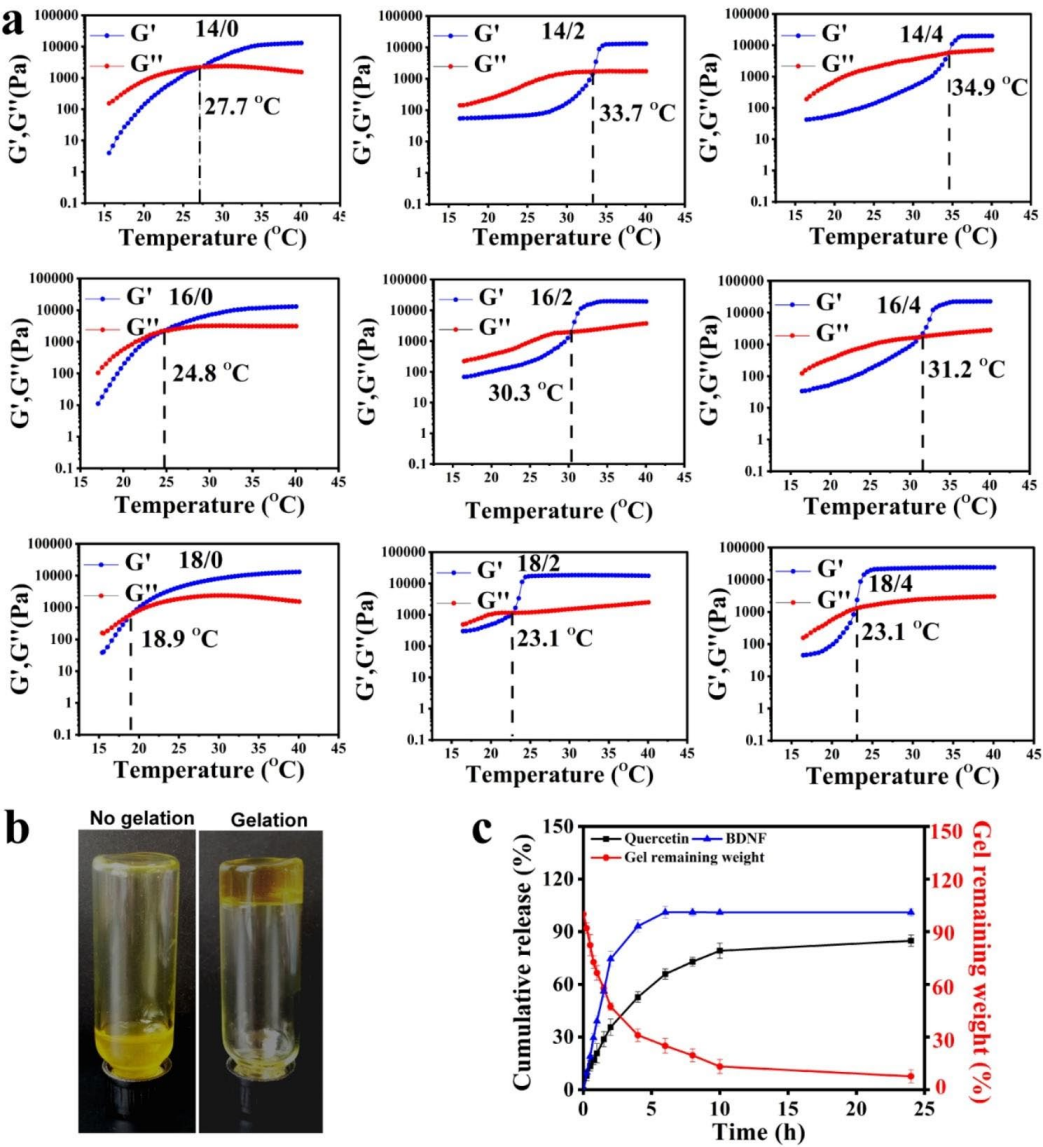
The characterization of BDNF-Quercetin nanogels in the thermosensitive gel. **(a)** Rheological behavior of quercetin nanogels in the thermosensitive gel (P 407 / P 188, w/w). **(b)** The appearance of quercetin nanogels in the thermosensitive gel. **(c)** Cumulative release profiles from BDNF-Quercetin nanogels in the thermosensitive gel

According to our previous studies, the nanogels-based thermosensitive hydrogel facilitates the improvement of the antidepressant effects of the drug by immobilizing the therapeutic agents locally in the nasal cavity and releasing them continuously [29, 30]. Hence, the temperature-sensitive property of quercetin nanogels in the thermosensitive gel was investigated by rheological analysis, which indicated the effects of poloxamer 407 (P 407) and P 188 on gelling temperature. The loss modulus (G’’) dominated storage modulus (G’), which suggested that thermosensitive gel was flowing, conversely forming an entangled network and viscous-like gels. For quercetin nanogels in the thermosensitive gel, the loss and storage modulus had the same modulus as gelling temperature, such that G′ was almost equal to G″ [31]. It was observed in Fig. 3a that gelling temperature with increased P 407 had a decreased tendency, but P 188 was the opposite. The G′ at about 30 °C presented a dramatic decrease, indicating the solution-gelation transition of quercetin nanogels in the thermosensitive gel. Gelation temperature adapted to intranasal delivery when P 407 and P 188 closed to the ratio of 16 to 2, approximately 30.3 ºC. Quercetin nanogels in the thermosensitive gel presented a flowing state at 25 ℃, whereas a non-flowing form at 37 ℃ (Fig. 3b**).**

The release behaviors of BDNF-Quercetin nanogels in the thermosensitive gel were investigated by PBS (pH 7.4). Cumulative release profiles and fitting analysis were indicated in Fig. 3c and Table S1, respectively. The release kinetics of BDNF-Quercetin nanogels were analyzed by computing the kinetic constant. The release kinetics of quercetin and BDNF were found to obey the first-order release curve, following the Ritger-Peppas model. These results indicated that the remaining BDNF-Quercetin nanogels in the thermosensitive gel slowly decreased as BDNF and quercetin were released sustainably with non-Fick’s diffusion. Until 24 h, BDNF-Quercetin nanogels in the thermosensitive gel were thoroughly degraded, and both components were released entirely from the nanogels.

### Cell biology evaluation and immune response

The in vitro cell viability was evaluated using Calcein-AM labeled fluorescence assay to assess lipopolysaccharide (LPS)-activated and normal RAW264.7 cells treated with quercetin, blank nanogels, and quercetin nanogels (Figure S2a). The enlarged cell volume and the changed cell morphology were observed after LPS stimulation compared with the control. Quercetin, blank nanogels, and quercetin nanogels had no cytotoxic effects compared to the control and exhibited nonsignificant effects on cell viability. In Figure S2b, the proliferative effects of quercetin nanogels on RAW264.7 cells growing were analyzed by the manufacturer’s instructions of the BrdU ELISA kit. The results indicated that quercetin, blank nanogels, and quercetin nanogels had no significant effects on the proliferation of RAW264.7 cells until 24 h.

Immune cells can produce some cytokines essential to regulating the immune response. Still, abnormal cytokines could lead to immune-mediated disorders, including allergies, infectious diseases, cancers, and autoimmunity. The previous study showed that examining the effects of nanogels on cytokine levels was worthwhile for immune systems induced by immune stimulators [32]. Natural active compounds have been widely used in treating diseases due to their low toxicity and good biocompatibility [33–35]. To investigate whether quercetin nanogels had immunostimulatory effects, TNF-α and IL-6 concentrations in the cell supernatant were determined after incubation with RAW 264.7 cells for 48 h. Compared with the control, quercetin nanogels and blank nanogels did not stimulate RAW 264.7 cells to secrete excessive inflammatory factors (Figure S2c-d), indicating that the quercetin nanogels and blank nanogels did not activate the cellular immune response.

### Evaluation of in vitro anti-inflammatory activities

The anti-inflammatory activity of quercetin nanogels is evaluated by LPS-induced RAW 264.7 cells. It was found that compared with blank nanogels, quercetin nanogels effectively restricted inducible nitric oxide synthase (iNOS) mRNA expression, further inhibiting overproduction of nitric oxide (NO) that the iNOS produced, and finally curbing the generation of nitrite, one of the products of nitric oxide (Figure S3a-c). These results showed that quercetin nanogels enhanced cell survival and might have effects on the outcome of depressive disorder caused by inflammation, consistent with previous studies [36]. Compared to the LPS group, quercetin nanogels down-regulated the expression of iNOS, NO, and nitrite, better than pure quercetin. Meanwhile, cytokines, the important biomarkers of nanocarrier immunotoxicity, have been widely used in immunotoxicity studies [37]. In particular, the secretion of TNF-α and IL-6 in RAW 264.7 cells increased in response to inflammation, playing a pivotal role in decreasing inflammation levels. As shown in Figure S3d-g, LPS up-regulated the mRNA and protein expression of TNF-α and IL-6 in RAW 264.7 cells. However, quercetin and quercetin nanogels significantly inhibited TNF-α and IL-6 mRNA expression and protein expression, with quercetin nanogels outperforming quercetin.

Generally, cyclooxygenase-2 (COX-2) activity in normal tissue cells was deficient, whereas COX-2 level in inflammatory cells would increase several times when inflammation stimulated cells. This elevation in prostaglandin E_2_ (PGE_2_), in turn, exacerbates the inflammatory response and induces tissue damage. Both COX-2 and PGE_2_ play pivotal roles in this inflammatory process. Compared with the LPS group, mRNA expression levels and protein expression of COX-2 and its downstream product, PGE_2_, were significantly up-regulated in LPS-induced RAW264.7 cells (Figure S3h-j). Conversely, both quercetin and quercetin nanogels showed significant inhibitory effects on mRNA expression and protein expression of COX-2 and PGE_2_ content. While blank nanogels did not exhibit significant anti-inflammatory effects compared to the LPS group, quercetin nanogels markedly inhibited the COX-2/PGE_2_ signaling pathway response. Overall, quercetin nanogels, compared to free quercetin, effectively inhibited the over-expression of inflammatory genes, including iNOS, IL-6, TNF-α, COX-2, and PGE_2_. Quercetin nanogels demonstrate excellent biocompatibility and augmented anti-inflammatory properties, thus positioning them as promising nanocarriers for treating depression and other inflammation-associated diseases.

### In vivo biodistribution and pharmacokinetics studies

**Fig. 4:**
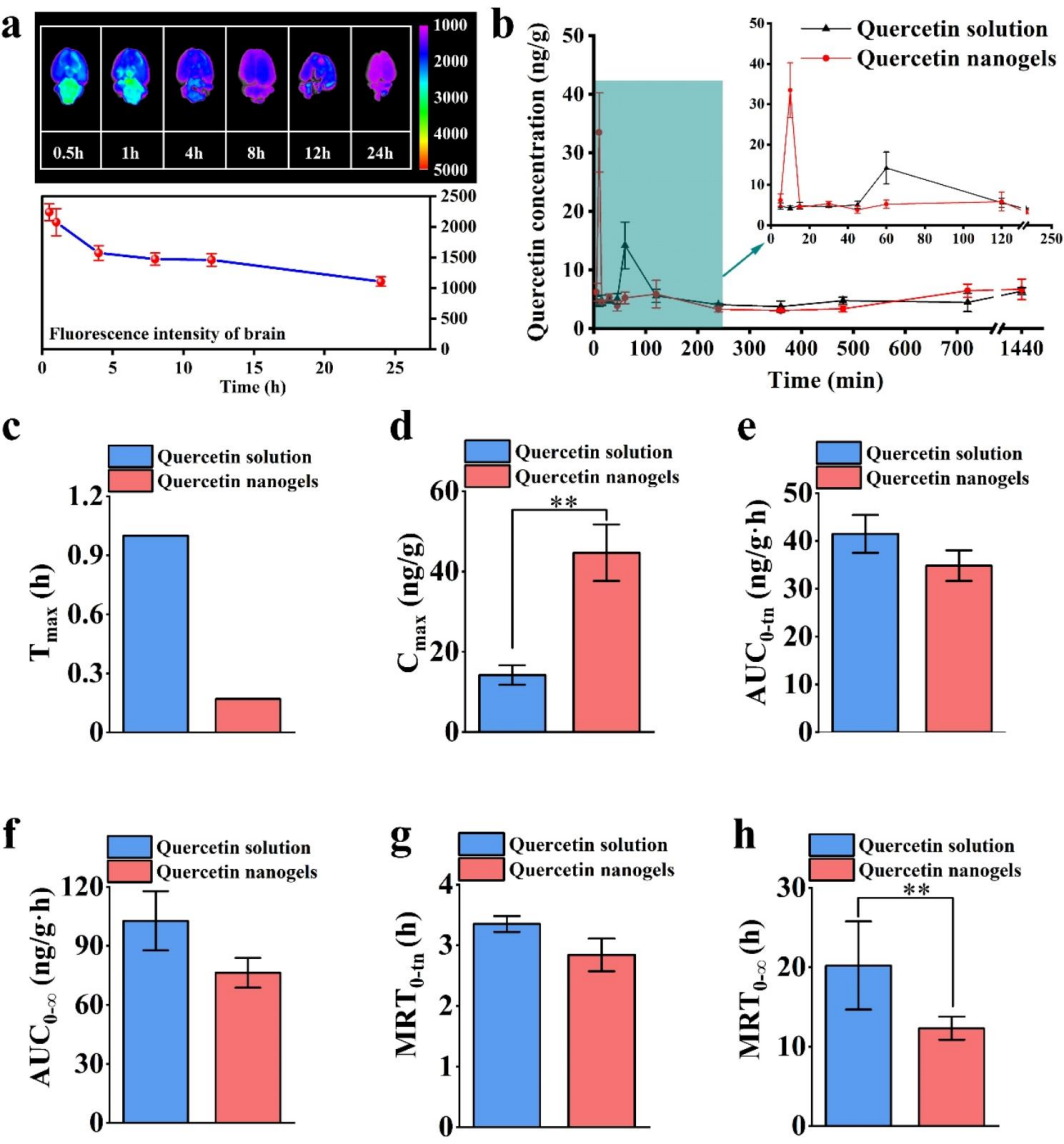
Distribution in brain and pharmacokinetic study of quercetin nanogels. **(a)** Fluorescence analysis of quercetin nanogels in the brain. **(b)** Hippocampal quercetin concentration-time curves after intragastric or intranasal delivery. Pharmacokinetic parameters include **(c)** T_max_ (h), **(d)** C_max_ (ng/g), **(e)** AUC_0 − tn_ (ng/g·h), **(f)** AUC_0−∞_ (ng/g·h), **(g)** MRT_0−∞_(h), **(h)**, MRT_0 − tn_**(h)**. ^**^p < 0.01 compared with the orally administrated quercetin solution

In vivo biodistribution and pharmacokinetics of quercetin nanogels were evaluated using Sprague-Dawley (SD) rats. Brain tissues and plasma analysis were performed at different periods after intranasal administration of quercetin nanogels. To assess their brain biodistribution, rhodamine B isothiocyanate (RBITC)-labeled quercetin nanogels were administrated, and the fluorescence was analyzed. It was found in Fig. 4a that the most intense fluorescence in the rat brain was observed approximately 30 min post-administration, followed by a gradual decrease. Quercetin nanogels were quickly delivered to the brain region and predominantly accumulated in the brain at short time scales, suggesting their potential for targeted brain delivery via the intranasal route, which could be beneficial for treating depressive disorders. To evaluate whether the quercetin nanogels improved in vivo bioavailability of quercetin, the corresponding plasma concentration vs. time **(**Fig. 4b**)** and pharmacokinetic parameters **(**Fig. 4c-h**)** of the orally administrated quercetin and intranasally administrated quercetin nanogels were analyzed, respectively. The concentration vs. time curve of quercetin nanogels showed a sharp increase at approximately 15 min. In contrast, oral administration of the quercetin reached a maximum plasma concentration of 4 h post-administration, resulting in a slower increase than quercetin nanogels. The intranasally administered quercetin nanogels exhibited a shorter T_max_ and higher C_max_ than orally administered quercetin, indicating rapid achievement of peak drug concentration. Furthermore, the bioavailability of the intranasally administered quercetin nanogels was nearly 50 times greater than that of orally administered quercetin, as determined by relative bioavailability calculations. Therefore, the quercetin nanogels provide the potential to overcome BBB and enhance quercetin bioavailability at lower doses.

### The antidepressant activities of quercetin nanogels and their BDNF delivery

**Fig. 5:**
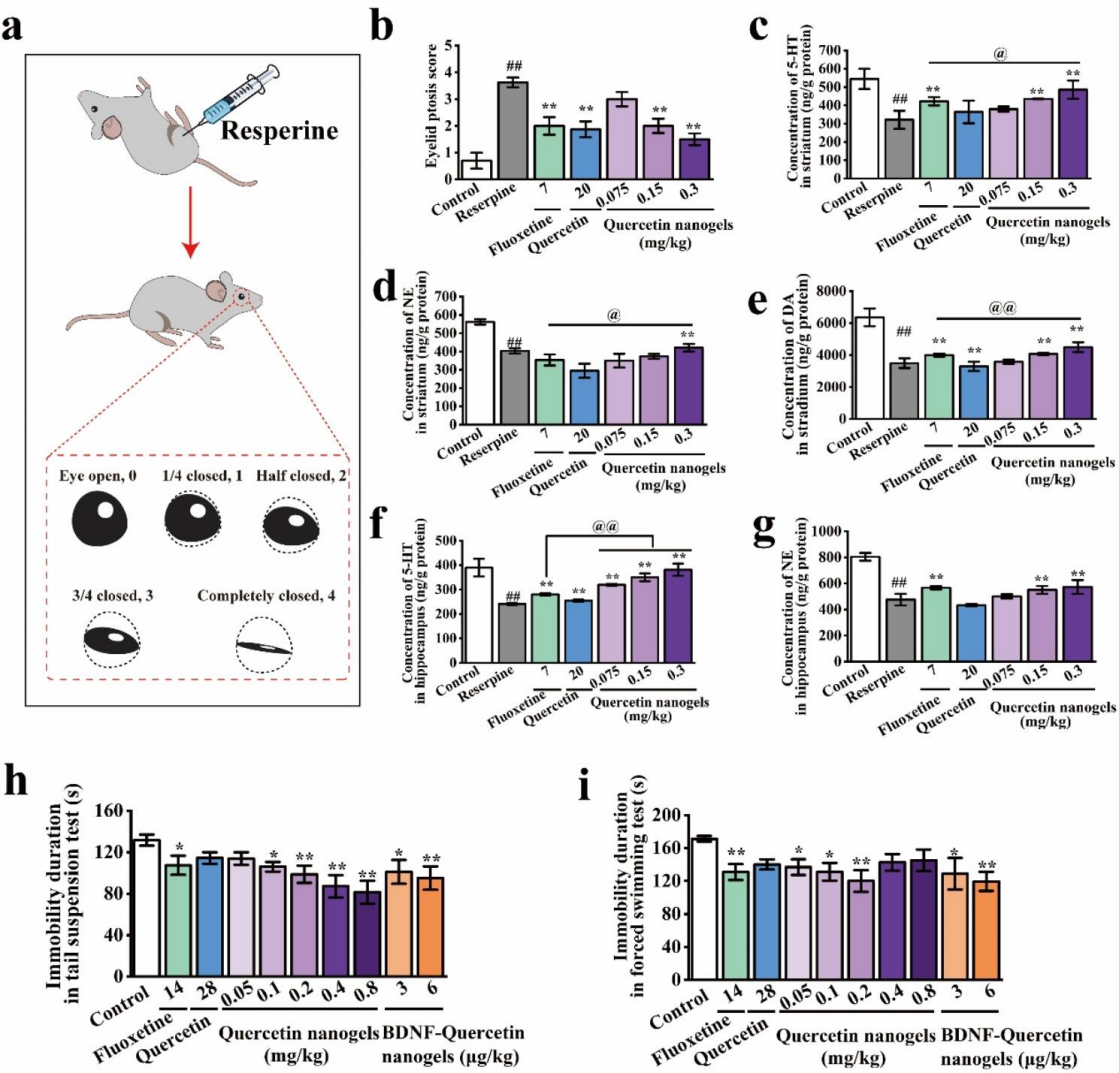
The antidepressant activities of quercetin nanogels and their BDNF delivery. **(a)** Schematic diagram of ptosis score of rats reduced by reserpine. **(b)** Eyelid ptosis score of rats. **(c)** 5-HT concentration in the striatum. **(d)** NE concentration in the striatum. **(e)** DA concentration in the striatum. **(f)** 5-HT concentration in the hippocampus. **(g)** NE concentration in the hippocampus. **(h)** Immobility duration of mice in tail suspension test. **(i)** Immobility duration of mice in a forced swimming test. ^##^*p* < 0.01 compared with the control group. ^*^p < 0.05, ^**^p < 0.01 compared with the reserpine group. ^@^*p* < 0.05, ^@@^*p* < 0.01 compared with fluoxetine

To investigate the potential antidepressant activities of quercetin nanogels, we tested the hypotheses using two models that contained intraperitoneal injection of reserpine and a well-established behavioral despair model. The research findings indicated that reserpine’s mechanism of action involved the depletion of biogenic amines. A substantial dose of reserpine resulted in the depletion of noradrenaline, adrenaline, dopamine, and 5-HT in the brain for a duration exceeding 7 days. However, depressive behavior persisted for only 3 days [38]. Concurrently, the reserpine-induced model was chosen due to its straightforward procedure and high success rate, making it a suitable choice for evaluating the therapeutic efficacy of antidepressants. A schematic diagram of the ptosis score of rats reduced by reserpine was indicated in Fig. 5a. It was used to assess the rats’ depression-like state induced by reserpine. The results suggested that the eyelid ptosis score of the administrated drugs was significantly lower than the model group induced by reserpine. Also, quercetin nanogels significantly alleviated the depletion of 5-HT, NE, and DA in the striatum and hippocampus **(**Fig. 5b-g**)**, suggesting that quercetin nanogels effectively restrained on reserpine-induced depletion of monoamine neurotransmitters.

Behavioral tests are used to assess the behavioral characteristics after intranasal administration, including the open field test (OFT), tail suspension test (TST), and forced swimming test (FST). The OFT was used to assess overall activity, anxiety-related behavior, and locomotor activity in a novel environment to rule out any inhibitory or excitatory effects of BDNF-quercetin nanogels [39]. The TST and FST were widely performed to investigate antidepressant activities and depression-like behavior of antidepressant drugs [40, 41]. In the OFT of the mice, the significant differences were not discovered in the rearing times and total distance, as shown in Figure S4a-b, compared to the control group, suggesting that the drugs had no impact on the tested mice. In the TST and FST (Fig. 5h-i), low doses of quercetin nanogels and BDNF-Quercetin nanogels significantly reduced the immobility duration, thereby improving depressive behavior and demonstrating superior antidepressant effects compared to other groups.

A preliminary study using two models manifested that BDNF-Quercetin nanogels had better antidepressant activities and were almost equal to the orally administrated fluoxetine and quercetin. Still, a dose of the former was lower, showing their superiority of brain targeting by intranasal delivery.

### Antidepressant effects of BDNF-Quercetin nanogels in the thermosensitive gel on the chronic unpredictable mild stress (CUMS) rats

**Fig. 6:**
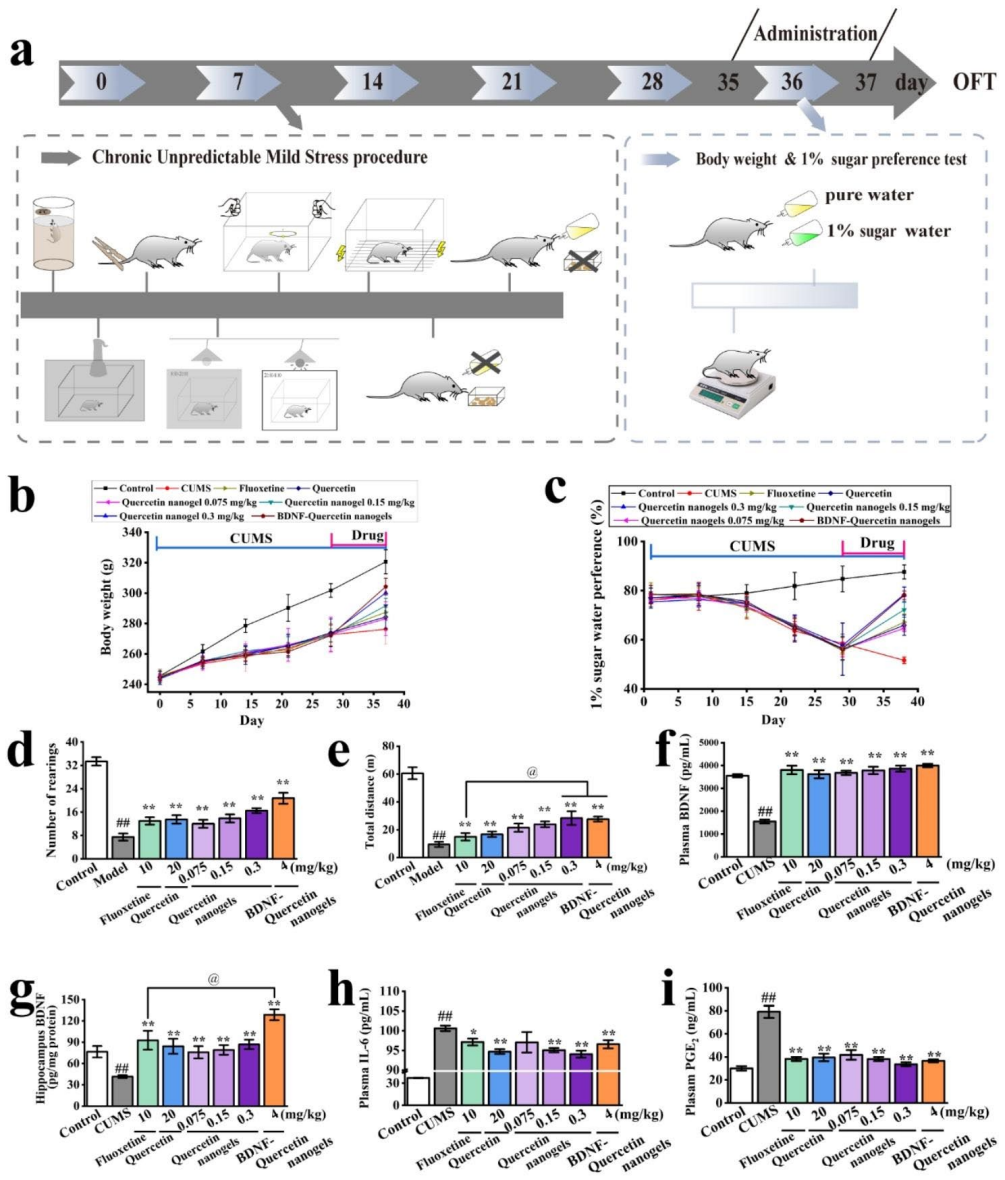
Antidepressant effects of BDNF-Quercetin nanogels on the CUMS rats. **(a)** Schematic diagram of the CUMS procedure. **(b)** Changes in body weight of rats. **(c)** Changes of 1% sucrose preference of rats. **(d)** Number of rearings. **(e)** Total distance of rats. **(f)** BDNF concentration in plasma. **(g)** BDNF content in the hippocampus. **(h)** IL-6 concentration in plasma. **(i)** PGE_2_ content in the hippocampus. ^##^*p* < 0.01 compared with the control group. ^*^p < 0.05, ^**^p < 0.01 compared with CUMS model. ^@^*p* < 0.01 compared with the fluoxetine

The CUMS model **(**Fig. 6a**)** was further performed to study the antidepressant mechanism of BDNF-Quercetin nanogels after the initial evaluation of antidepressant activity using two animal models. It was observed that CUMS-induced weight loss and anhedonia were substantially mitigated by the experimental drugs, particularly BDNF-Quercetin nanogels, demonstrating a better effect on the stressed rats **(**Fig. 6b-c**)**. The OFT was used to assess the exploratory behavior of the drug-treated CUMS rats after administration. It was indicated that fluoxetine, quercetin, quercetin nanogels, and BDNF-quercetin nanogels increased the number of rearings and the total distance of CUMS model **(**Fig. 6d-e**).** Both BDNF-quercetin nanogels and quercetin nanogels demonstrated more significant antidepressant effects than fluoxetine, as evidenced by the total distance. Plasma and hippocampal BDNF concentrations of the CUMS rats were markedly enhanced by the experimental drugs. Notably, the hippocampal BDNF level in the BDNF-Quercetin nanogel group exhibited a significant change compared to the fluoxetine group. These findings suggested that the delivery of exogenous BDNF compensated for the loss of BDNF in the brain (Fig. 6f-g), thereby enhancing its antidepressant effects.

Clinical reports and meta-analyses found that depressed patients had significant hypothalamic-pituitary-adrenal (HPA) axis hyperactivation. These dysfunctions increased the stress hormone cortisol secretion and inflammatory biomarkers’ levels [42–46]. It was shown in Figure S5a-c that the levels of corticotropin-releasing hormone (CRH), adrenocorticotropic Hormone (ACTH), and corticosterone in rats were significantly increased by CUMS. However, the only level of corticosterone was decreased considerably by BDNF-Quercetin nanogels compared to CUMS rats. Many studies have found that testosterone had antidepressant effects in socially isolated male but not female rats [47]. It was observed in Figure S5d that quercetin nanogels, in a dose-dependent manner, improved the testosterone level, and BDNF-Quercetin nanogels also showed a significant increase compared to CUMS rats. In the meantime, inflammation was a crucial biological event that might increase the risk of major depressive disorders. CUMS-induced rats exhibited higher IL-6 and PGE_2_ levels in plasma **(**Fig. 6h-i**)**. Inflammatory levels of the rats were decreased after administration. BDNF-Quercetin nanogels also presented significant differences compared to those without administration. In summary, BDNF-Quercetin nanogels at a lower dose decreased the abnormal behavior of CUMS model and improved their biochemical indicators.

**Fig. 7:**
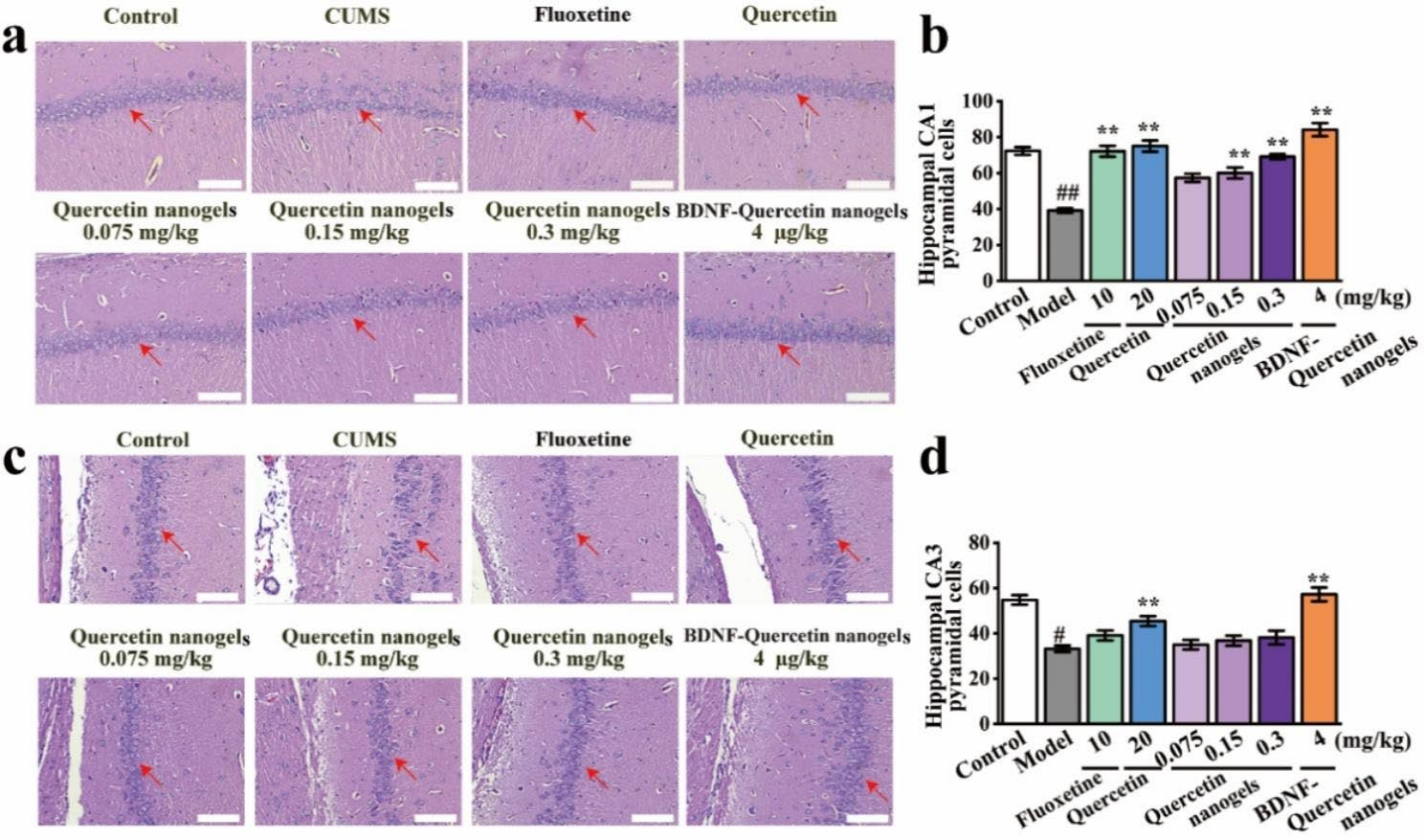
Pathological changes in rat hippocampal tissues. **(a)** Pathological changes of CA1 subregion in rat hippocampal tissues (scale bar: 100 μm). **(b)** Hippocampal CA1 pyramidal cells. **(c)** Pathological changes of CA3 subregion in rat hippocampal tissues (scale bar: 100 μm). **(d)** Hippocampal CA3 pyramidal cells. ^##^*p* < 0.01 compared with the control group. ^*^p < 0.05, ^**^p < 0.01 compared with CUMS model

**Fig. 8:**
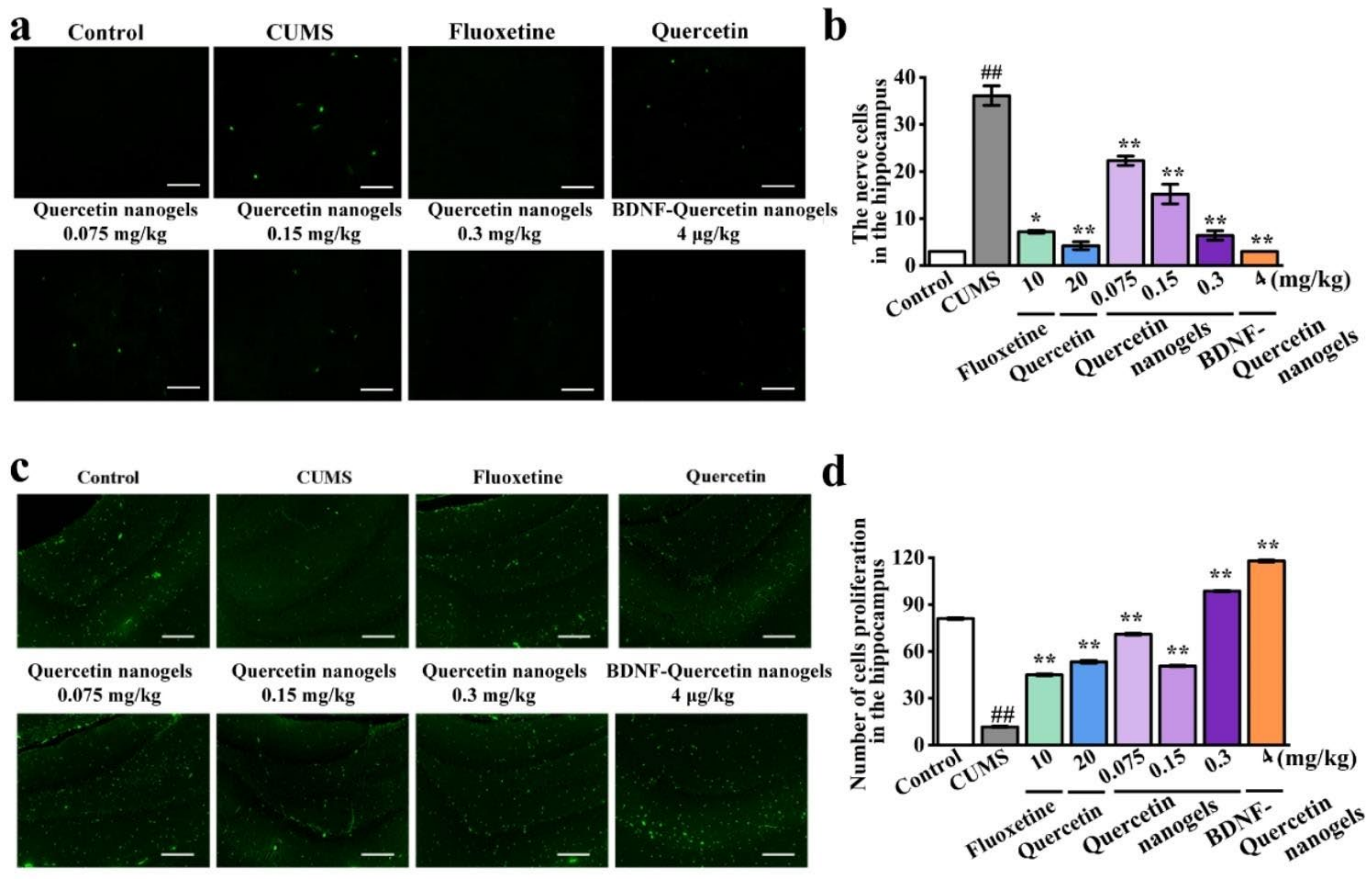
CUMS-induced cell apoptosis and proliferation in the hippocampus of rats. **(a)** TUNEL staining. **(b)** The nerve cells by TUNEL staining. **(c)** Ki67 staining. **(d)** Number of cell proliferation by Ki67 staining. ^##^*p* < 0.01 compared with the control group. ^*^p < 0.05, ^**^p < 0.01 compared with the CUMS model

Effects of BDNF-Quercetin nanogels on rat hippocampal pyramidal cells were investigated. It was observed in the H&E staining that there was dramatic damage to pyramidal cells in the hippocampal subregion of CUMS rats (Fig. 7a-d). Compared to CUMS model, these abnormalities were significantly improved by BDNF-Quercetin nanogels. Similarly, cell apoptosis and proliferation were also observed and analyzed by TUNEL and Ki67 staining, respectively. The number of positive cells in CUMS rats significantly decreased (Fig. 8a-d). BDNF-Quercetin nanogels significantly increased the corresponding positive cells. At the same time, these impairments of pyramidal cells were also found in the prefrontal cortex of CUMS rats (Figure S6), and BDNF-Quercetin nanogels showed the same therapeutic effects as in the hippocampus.

Apoptosis is an essential mechanism of CUMS-induced depression [48]. As an anti-apoptotic endogenous membrane protein, B cell lymphoma-2 (Bcl-2) prevents cells from entering the apoptotic program. B-cell lymphoma extra-large (Bcl-xL), like Bcl-2, is another antiapoptotic factor supporting neuronal survival but not promoting axon regeneration. BCL2-associated X (Bax) was a pro-apoptotic protein promoting cells to enter apoptosis. The study findings indicated that CUMS increased Bax mRNA expression in the hippocampal and prefrontal cortex tissues (Figure S7a, b) and selectively decreased hippocampal Bcl-xL mRNA levels (Figure S7c, d) without changing Bcl-2 mRNA expression (Figure S7e, f).

BDNF-quercetin nanogels exerted antidepressant effects on CUMS rats, mainly through anti-stress, anti-inflammation, and neuroprotection. BDNF-quercetin nanogels can restore HPA axis function, inhibit neuron cell apoptosis, and promote neuron cell regeneration. To elucidate the impact of BDNF-Quercetin nanogels on CUMS rats, a comprehensive, integrated omics approach was used to investigate potential antidepressant mechanisms.

### Antidepressant mechanism of BDNF-Quercetin nanogels in the thermosensitive gel on the CUMS rats

**Fig. 9:**
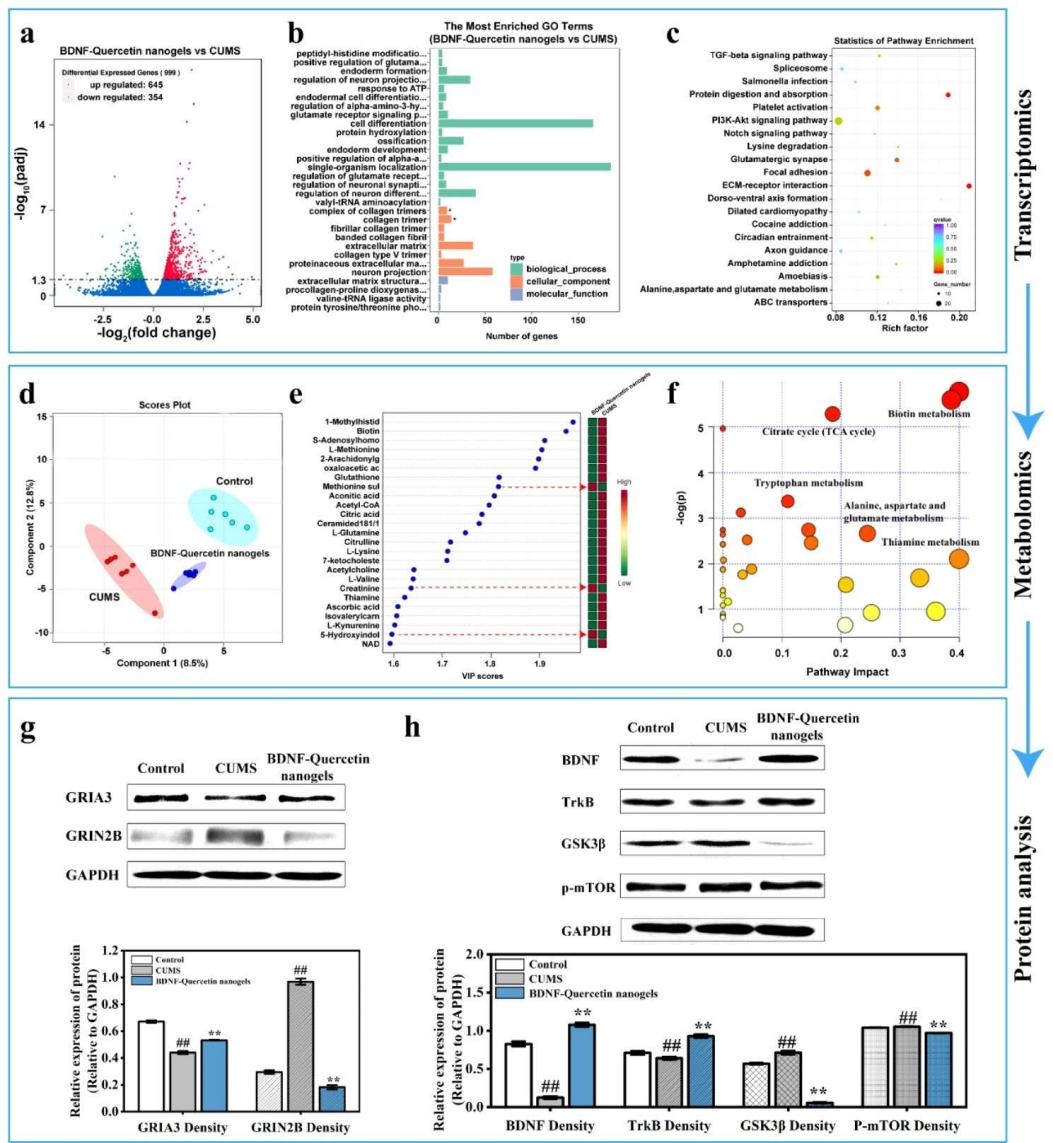
Antidepressant mechanism of BDNF-Quercetin nanogels in the thermosensitive gel on the CUMS rats. **(a)** Volcano map. **(b)** GO enrichment analysis. **(c)** KEGG pathway enrichment analysis of BDNF-quercetin nanogels vs. CUMS model. **(d)** Partial least squares-discriminant analysis (PLS-DA). **(e)** VIP analysis. **(f)** Major metabolic pathways that BDNF-Quercetin nanogels improve CUMS rats. **(g-h)** Expression of GRIA3, BDNF, TrkB, P-mTOR, GRIN2B, and GSK3β in the hippocampus of rats. ^##^*p* < 0.01 compared with the control group. ^**^p < 0.01 compared with the CUMS group

Gene expression of total mRNA isolated from rat hippocampus was assessed using RNA sequencing. The transcriptomic volcano map revealed that compared to CUMS group, 999 genes were differentially expressed in the BDNF-quercetin nanogels group, with significantly up-regulated 645 genes and significantly down-regulated 354 genes **(**Fig. 9a**)**. The most enriched GO terms in Fig. 9b presented that BDNF-quercetin nanogels changed biological processes (including peptidyl-histidine modification and positive regulation of glutamate receptor), cellular components (including a complex of collagen trimers), and molecular function (extracellular matrix structural constituent) of CUMS rats. Gene set enrichment analysis (GSEA) was next performed using the KEGG (Kyoto Encyclopedia of Genes and Genomes) database, revealing significant enrichment including TGF-β and PI3K-Akt signaling pathway, glutamatergic synapse, alanine, aspartate, and glutamate metabolism **(**Fig. 9c**)**. Additionally, GO terms associated with antioxidant activity were identified in **Table S4**. Oxidative phosphorylation was further found in KEGG pathway enrichment analysis. In line with previous studies on depression, it was found that there are glutamate receptor-related terms, PI3K-Akt, glutamatergic synapse, and glutamate metabolism signaling pathways. These transcriptomic findings suggested that BDNF-Quercetin nanogels not only induced antioxidant activities via oxidative phosphorylation but also exerted antidepressant effects on CUMS rats by improving the glutamatergic system and PI3K-Akt signaling pathway. A metabolomic analysis was then performed to investigate the changes in hippocampal metabolites. Partial least square analysis (PLS-DA) in Fig. 9d indicated that the influence of BDNF-Quercetin nanogels on metabolites neared the control and differed from CUMS group, showing that control, CUMS, and BDNF-Quercetin nanogels had a significant difference in the levels of total metabolites. Further analysis in Fig. 9e showed that compared to CUMS group, some metabolites (including methionine sulfoxide, creatinine, 5-Hydroxyindoleacetic acid) were up-regulated, while others (including 1-Methylhistidine, biotin, 2-Arachidonylglycerol, S-Adenosylhomocysteine, Acetyl-CoA, 7-ketocholesterol, Ascorbic acid, L-Methionine) were down-regulated. The metabolic pathways associated with the antidepressant effects primarily encompassed the following biochemical process: (1) biotin metabolism; (2) citrate cycle; (3) tryptophan metabolism; (4) Alanine, aspartate, and glutamate metabolism; (5) thiamine metabolism **(**Fig. 9f**)**. Taken together, transcriptomic and metabonomic data showed that the glutamatergic system and PI3K-Akt signaling pathway were the primary antidepressant mechanism of BDNF-Quercetin nanogels in CUMS rats.

Western blot experiments were performed to verify related protein expression. Protein expression of the glutamatergic system was demonstrated in Fig. 9g. These results presented that chronic stress significantly reduced the GRIA3 (the receptor of AMPA) protein content and increased the GRIN2B (the receptor of NMDA) protein content in the hippocampus of rats. Moreover, BDNF-Quercetin nanogels regulated the PI3K-Akt signaling pathway by improving the abnormal expression of BDNF, TrkB, GSK3β, and p-mTOR after CUMS **(**Fig. 9h**)**. These results showed that exogenously supplemented BDNF might bind to its receptor TrkB and further activate the PI3K-Akt signaling pathway, thereby regulating the expression of abnormal GSK3β and p-mTOR proteins.

In summary, the protective effects of quercetin nanogels on BDNF were achieved by antioxidant activities associated with oxidative phosphorylation. By integrating omics prediction and protein expression verification, the results showed that BDNF-Quercetin nanogels exerted antidepressant effects on CUMS rats by modulating the glutamatergic system and PI3K-Akt signaling pathway.

## Conclusion

In conclusion, quercetin nanogels were successfully prepared and characterized. Quercetin nanogels showed protective effects against protein damage, exhibited antioxidant activities without affecting cell viability, proliferation, and immune response, and increased in vitro inflammatory cytokine levels. The intranasally administered quercetin nanogels rapidly distributed in the brain within 30 min and improved the bioavailability of quercetin nearly 50-fold at a lower dose. The BDNF-quercetin nanogels in the thermosensitive gel exhibited excellent thermosensitivity and co-delivered quercetin and BDNF slowly and sustainably. As a protein drug carrier, quercetin nanogels exerted antidepressant effects on reserpine-induced rats and alleviated the depletion of monoamine neurotransmitters. BDNF-quercetin nanogels effectively reversed despair behavior in mice, alleviated weight loss and anhedonia in rats, ameliorated dramatic pyramidal cell damage in hippocampal CA1 and CA3 subregions and inflammatory cytokine levels, and ameliorated CUMS-induced cell apoptosis and proliferation in rat hippocampus. Further omics analysis and protein verification revealed that the treatment of BDNF-Quercetin nanogels on depressive disorder was mainly related to the glutamatergic system, PI3K-Akt, and BDNF-TrkB signaling pathway. These results showed that the brain delivery of BDNF- Quercetin nanogels via intranasal administration has a significant potential for the combination treatment of depressive disorder.

## Experimental section

Chemicals and Materials and animals are described in Additional file 1: S1 Chemicals and Materials and S2 Animals, respectively.

### Preparation and characterization of BDNF-Quercetin nanogels

BDNF-Quercetin nanogels were prepared using the previous study with slight modifications [49]. Preparation and characterization of BDNF-Quercetin nanogels are described in Additional file 1: S3 Preparation and characterization of BDNF-Quercetin nanogels.

In vitro release of quercetin nanogels was investigated in 0.1 M Tris-HCl (pH 7.4), 0.1 M Tris-HCl (pH 8.0), 0.1 M PBS (pH 7.4), 0.01 M Tris-HCl (pH 7.4), and 1 M Tris-HCl (pH 7.4). In vitro release of BDNF-Quercetin nanogels was investigated in 0.1 M Tris-HCl (pH 7.4), and 0.1 M PBS (pH 7.4). 25 mg of quercetin nanogels or BDNF-Quercetin nanogels were added to 2 mL of release medium to disperse them fully. Quercetin nanogels and BDNF-Quercetin nanogels were placed in a dialysis bag (molecular weight of 10,000) and suspended in 40 mL of release medium at 34 °C on a shaker at 50 rpm. Each release sample was taken at appropriate intervals and added by fresh release medium. The analysis was conducted three times for each batch.

*Antioxidant activities of quercetin nanogels*: The protective effects of quercetin on protein were determined using the method of Coomassie brilliant blue. The methods are described in the Additional file 1: S4 Antioxidant activities of quercetin nanogels.

### Preparation and characterization of BDNF-Quercetin nanogels in the thermosensitive gel

BDNF-Quercetin nanogels in the thermosensitive gel were obtained using two steps. One step was that BDNF-Quercetin nanogels were dissolved in normal saline at room temperature, cooling to 4 °C. Two-step was that the pre-made BDNF-Quercetin nanogels solution was mixed with P 407 and P 188 in different proportions (14/0, 14/1, 14/2, 16/0, 16/1, 16/2, 18/0, 18/1, 18/2, w/w) to form a stable BDNF-Quercetin nanogels stock solution at 4 °C.

The rheological measurements of BDNF-Quercetin nanogels in the thermosensitive gel were performed to analyze the effects of P 407/ P188 ratio on its solution–gel–solution-phase transition by Anton-Paar rheometer (MCR 302, Austria). 0.5 mL stock solution was placed between parallel plates with a gap of 1 mm. The storage modulus (G’) and the loss modulus (G’’) were measured under 1 Hz and shear stress of 0.01 Pa, ranging from 15 to 40 °C. The gelation temperature was observed through changes in the modulus [50].

In vitro release of BDNF-Quercetin nanogels in the thermosensitive gel was explored using PBS to mimic intranasal release behavior. BDNF-Quercetin nanogels in the thermosensitive gel (P 407/ P188, 16/2) containing 16% of BDNF-Quercetin nanogels were added into the vial (2.7 cm x 4.7 cm) with heating at 37 ºC for 10 min in the incubator shaker until phase transition from solution to gel and then were soaked in 2 mL PBS (pH 6.4) with gently shaking (55 rpm). Each release sample was taken at appropriate intervals and added to fresh PBS. The residual weight was weighed and compared with the initial weight to calculate the weight loss rate. Each batch was analyzed in triplicate.

### Cell culture

RAW 264.7 cells were cultured in high-glucose DMEM containing phenol red, 10% heated-inactivity fetal bovine serum (HI-FBS), 100 U/mL penicillin, and 100 µg/mL streptomycin at 37 °C in a humidified 5% CO_2_ air. A stable cell line was established and maintained in a medium supplemented with high-glucose DMEM containing 5% HI-FBS without phenol red. Cell biology evaluation and immune response were performed in Additional file 1: S5 Cell biology evaluation and immune response.

### Distribution in the brain and pharmacokinetic study

For brain distribution studies, the Sprague-Dawley (SD) rats, intranasally administered to RBITC-labeled quercetin nanogels in the thermosensitive gel, were investigated after being sacrificed for 0.5 h, 1 h, 4 h, 8 h, 12 h, and 24 h. The rats’ brain was removed and then observed by a CRI fluorescence imaging system (Maestro 2, CRI, USA). In a pharmacokinetic study, SD rats were randomly divided into intranasal (quercetin nanogels in the thermosensitive gel) and oral administration (quercetin solution) at the dose of 0.3 and 20 mg/kg (calculated by free quercetin dose) body weight, respectively. The hippocampi were collected, detected, and analyzed as described in Additional file 1: S6 Pharmacokinetic study.

### Animal experiments

Open field test (OFT), forced swim test (FST), tail suspension test (TST), and reserpine-induced depression model are described in Additional file 1: S7 Open field test (OFT) and forced swim test (FST), tail suspension test (TST), and reserpine-induced depression model. The chronic unpredictable mild stress (CUMS) model was used to investigate the antidepressant effect of BDNF-Quercetin nanogels in the thermosensitive gel and its mechanism. The protocol was approved by the Animal Ethics Committee of Tianjin University of Traditional Chinese Medicine (TCM-2016-038-E14). The experiments are reported by the Animal Research: Reporting in Vivo Experiments (ARRIVE) guidelines. The SD rats were divided into control and CUMS groups treated with drugs. CUMS involved exposure to various mild stressors. After the last 7 days of CUMS, the rats were administrated, which included CUMS, fluoxetine (10 mg/kg, intragastric administration), quercetin (20 mg/kg, intragastric administration), quercetin nanogels in the thermosensitive gel (0.075, 0.15, 0.3 mg/kg calculated by the weight of quercetin, intranasal administration), BDNF-Quercetin nanogels in the thermosensitive gel containing BDNF (4 µg/kg) and quercetin (0.15 mg/kg) via intranasal administration. After administration, the locomotor activity was evaluated by the OFT, consisting of the number of rearings and total distance. The rat heparinized plasma was collected by centrifugation at 3000 × g for 10 min after the behavioral despair test. According to the manufacturer’s instructions, the change of BDNF content in plasma and hippocampi was detected by the BDNF Emax® Immunoassay System. The inflammatory levels (TNF-α and IL-6) and HPA axis function (CRH, ACTH, corticosterone, and testosterone) were measured by ELISA Assay Kit. The hippocampi of the rats were removed and then fixed in 10% neutral formalin or snap-frozen in liquid nitrogen for studying pathological changes, cell apoptosis, and cell proliferation in rat hippocampal tissues. For pathological research, the fixed hippocampus was dehydrated, embedded in paraffin, sectioned, and stained with hematoxylin and eosin (H&E). The CA1 and CA3 regions in the hippocampus were observed and photographed by a light microscope (OLYMPUS, Japan). To determine the end-stage apoptosis of hippocampal pyramidal neurons, TUNEL assays were performed on paraffin sections using a TUNEL apoptosis assay kit according to the manufacturer’s instructions. At the same time, the mRNA expression of the apoptosis gene was performed in a Bio-Rad C1000 (Bio-Rad, Pleasanton, CA, USA). The real-time RT-PCR primers of GAPDH, Bcl-2, Bax, and Bcl-xL were demonstrated in **Table S3**. The folds increase or decrease was calculated relative to blank control after normalized to a housekeeping gene using the 2^−ΔΔCT^ method. To measure pyramidal neuron proliferation in the hippocampus, the slides were incubated with an anti-MKI67 polyclonal antibody overnight at 4 °C, then a secondary antibody anti-rabbit IgG. The number of positive cells was counted using a Nikon Eclipse Ti-U inverted fluorescent microscope (Nikon, Japan).

The antidepressant mechanism of BDNF-Quercetin nanogels was explored by combining RNA sequencing, metabolomic analysis, and Western blot analysis. Transcriptome sequencing was accomplished by Beijing Novogene Technology Co. Ltd, as presented in Additional file 1: S8 The procedure of RNA sequencing. Metabolite levels in the hippocampal tissues were determined using our previous methods in Additional file 1: S9 Determination of hippocampal tissues in metabolite levels. The protein expressions of GRIA3, GRIN2B, BDNF, TrkB, GSK3β, and p-mTOR were determined by western blotting according to Additional file 1: S10 Western blotting detection.

### Statistical analysis

Statistical analyses were calculated by the Origin Pro 2023 software (Origin Lab, Northampton, MA). Results were expressed as the mean ± standard error of the mean (SEM). Statistically significant differences between groups were evaluated by one-way analysis of variance (ANOVA), followed by Tukey’s post hoc test. A *p* < 0.05 was considered statistically significant.

### Electronic supplementary material

Below is the link to the electronic supplementary material.

Supplementary Material 1

## Data Availability

All data generated or analyzed during this study are included in this published article.

## References

[1] https://www.who.int/health-topics/depression (accessed 17 June 2023).

[2] Li W, Ali T, Zheng C, He K, Liu Z, Shah FA, Li N, Yu Z-J, Li S (2022). Anti-depressive-like behaviors of APN KO mice involve Trkb/BDNF signaling related neuroinflammatory changes. Mol Psychiatry.

[3] Carniel BP, da Rocha NS (2020). Brain-derived neurotrophic factor (BDNF) and inflammatory markers: perspectives for the management of depression. Prog Neuropsychopharmacol Biol Psychiatry.

[4] Woodburn SC, Asrat HS, Flurer JK, Schwierling HC, Bollinger JL, Vollmer LL, Wohleb ES (2023). Depletion of microglial BDNF increases susceptibility to the behavioral and synaptic effects of chronic unpredictable stress. Brain Behav Immun.

[5] Huang F, Chen T, Chang J, Zhang C, Liao F, Wu L, Wang W, Yin Z (2021). A conductive dual-network hydrogel composed of oxidized dextran and hyaluronic-hydrazide as BDNF delivery systems for potential spinal cord injury repair. Int J Biol Macromol.

[6] Ma X, Gao F, Su W, Ran Y, Bilalijiang T, Tuolhen Y, Tian G, Ye L, Feng Z, Xi J (2023). Multifunctional injectable hydrogel promotes functional recovery after Stroke by modulating microglial polarization, angiogenesis and neuroplasticity. Chem Eng J.

[7] Park SB, Cho H-J, Moon SR, Choi KJ, Jung WH, Kim KY, Koh B. Gold nanoparticle-assisted delivery of brain-derived neurotrophic factor to cerebral organoids. Nano Res. 2022:1–7.

[8] Kraus A, Huertas M, Ellis L, Boudinot P, Levraud J-P, Salinas I (2022). Intranasal delivery of SARS-CoV-2 spike protein is sufficient to cause olfactory damage, inflammation and olfactory dysfunction in zebrafish. Brain Behav Immun.

[9] Zhang L, Deng L, Ma C, Zhang H, Dang Y (2023). Brain-derived neurotrophic factor delivered Intranasally relieves post-traumatic stress disorder symptoms caused by a single prolonged stress in rats. Neuropsychobiology.

[10] Zhou X, Deng X, Liu M, He M, Long W, Xu Z, Zhang K, Liu T, So K-F, Fu Q-L (2023). Intranasal delivery of BDNF-loaded small extracellular vesicles for cerebral ischemia therapy. J Control Release.

[11] Long Y, Yang Q, Xiang Y, Zhang Y, Wan J, Liu S, Li N, Peng W (2020). Nose to brain drug delivery-a promising strategy for active components from herbal medicine for treating cerebral ischemia reperfusion. Pharmacol Res.

[12] Cristea IA, Naudet F (2019). US Food and Drug Administration approval of esketamine and brexanolone. Lancet Psychiatry.

[13] Kryst J, Kawalec P, Pilc A (2020). Efficacy and safety of intranasal esketamine for the treatment of major depressive disorder. Expert Opin Pharmacother.

[14] Quintana DS, Steen NE, Andreassen OA (2018). The Promise of Intranasal Esketamine as a Novel and effective antidepressant. JAMA Psychiatry.

[15] Kandemir K, Tomas M, McClements DJ, Capanoglu E (2022). Recent advances on the improvement of quercetin bioavailability. Trends Food Sci Technol.

[16] Onugwu AL, Nwagwu CS, Onugwu OS, Echezona AC, Agbo CP, Ihim SA, Emeh P, Nnamani PO, Attama AA, Khutoryanskiy VV (2023). Nanotechnology based drug delivery systems for the treatment of anterior segment eye Diseases. J Control Release.

[17] Liu W, Ma Z, Wang Y, Yang J (2023). Multiple nano-drug delivery systems for intervertebral disc degeneration: current status and future perspectives. Bioact Mater.

[18] Chen YB, Zhang YB, Wang YL, Kaur P, Yang BG, Zhu Y, Ye L, Cui YL (2022). A novel inhalable quercetin-alginate nanogel as a promising therapy for acute lung injury. J Nanobiotechnol.

[19] Correa D, Scheuber MI, Shan H, Weinmann OW, Baumgartner YA, Harten A, Wahl A-S, Skaar KL, Schwab ME. Intranasal delivery of full-length anti-Nogo-A antibody: A potential alternative route for therapeutic antibodies to central nervous system targets. Proc. Natl. Acad. Sci. 2023, 120:e2200057120. 10.1073/pnas.2200057120 PMC9942809 36649432

[20] Liu Z, Tang W, Liu J, Han Y, Yan Q, Dong Y, Liu X, Yang D, Ma G, Cao H (2023). A novel sprayable thermosensitive hydrogel coupled with zinc modified metformin promotes the healing of skin wound. Bioact Mater.

[21] Madry H, Gao L, Rey-Rico A, Venkatesan JK, Muller-Brandt K, Cai X, Goebel L, Schmitt G, Speicher-Mentges S, Zurakowski D (2020). Thermosensitive hydrogel based on PEO-PPO-PEO poloxamers for a controlled in situ release of recombinant Adeno-Associated viral vectors for effective gene therapy of cartilage defects. Adv Mater.

[22] Donati I, Christensen BE. Alginate-metal cation interactions: macromolecular approach. Carbohydr Polym. 2023:121280. 10.1016/j.carbpol.2023.121280 37739522

[23] Li X, Liu Y, Yu Y, Chen W, Liu Y, Yu H (2019). Nanoformulations of quercetin and cellulose nanofibers as healthcare supplements with sustained antioxidant activity. Carbohydr Polym.

[24] Tan Y, Zi Y, Peng J, Shi C, Zheng Y, Zhong J. Gelatin as a bioactive nanodelivery system for functional food applications. Food Chem. 2023:136265. 10.1016/j.foodchem.2023.136265 37167667

[25] Kumar MN, Kalarikkal SP, Bethi CM, Singh SN, Narayanan J, Sundaram GM (2023). An eco-friendly one-pot extraction process for curcumin and its bioenhancer, piperine, from edible plants in exosome-like nanovesicles. Green Chem.

[26] Huang K, Zhong P, Xu B (2023). Discrimination on potential adulteration of extra virgin olive oils consumed in China by differential scanning calorimeter combined with dimensionality reduction classification techniques. Food Chem.

[27] Guo S, Kim WJ, Lok J, Lee SR, Besancon E, Luo BH, Stins MF, Wang X, Dedhar S, Lo EH. Neuroprotection via matrix-trophic coupling between cerebral endothelial cells and neurons. Proc. Natl. Acad. Sci. U. S. A. 2008, 105:7582–7587. 10.1073/pnas.0801105105 PMC2396701 18495934

[28] Xu D, Hu MJ, Wang YQ, Cui YL (2019). Antioxidant activities of Quercetin and its complexes for Medicinal Application. Molecules.

[29] Xu D, Qiao T, Wang Y, Wang QS, Cui YL (2021). Alginate nanogels-based thermosensitive hydrogel to improve antidepressant-like effects of albiflorin via intranasal delivery. Drug Deliv.

[30] Xu D, Lu YR, Kou N, Hu MJ, Wang QS, Cui YL (2020). Intranasal delivery of icariin via a nanogel-thermoresponsive hydrogel compound system to improve its antidepressant-like activity. Int J Pharm.

[31] Zhang R, Liu F, Zhang Q, Yang L, Hou X, Du T, Fan J, Hu H, Deng H, Hao L. Intra-articular delivery system of methotrexate for rheumatoid arthritis therapy: an in-suit thermosensitive comprehensive gel of polysaccharide from Aconitum carmichaelii Debx. Int J Biol Macromol. 2023:124822. 10.1016/j.ijbiomac.2023.124822 37257527

[32] Zhu H, Kong B, Che J, Zhao Y, Sun L. Bioinspired nanogels as cell-free DNA trapping and scavenging organelles for rheumatoid arthritis treatment. Proc. Natl. Acad. Sci. 2023, 120:e2303385120. 10.1073/pnas.2303385120 PMC10438393 37549284

[33] Xiang K, He Q, Chen Y, Yang D, Duan Y, Li H, Chen L (2021). Chemical constituents isolated from the aerial parts of Swertia pseudochinensis and their potential neuroprotective effects. Acupunct Herb Med.

[34] Yang M, Oppong MB, Di J, Yuan Q, Chang Y, Jiang M, Cao S, Dong P, Li L, Xie Y (2022). Steroidal saponins with anti-inflammatory activity from Tribulus terrestris L. Acupunct Herb Med.

[35] Zuo X, Yao R, Zhao L, Zhang Y, Lu B, Pang Z (2022). Campanumoea javanica bl. Activates the PI3K/AKT/mTOR signaling pathway and reduces Sarcopenia in a T2DM rat model. Acupunct Herb Med.

[36] Xu J, Qiu W, Liang M, Ye M, Hu J, Ma X, Shi X, Xue P, Kang Y, Xiao B (2023). Dual-stimulus phototherapeutic nanogel for triggering pyroptosis to promote cancer immunotherapy. J Control Release.

[37] Liu MX, Xu L, Cai YT, Wang RJ, Gu YY, Liu YC, Zou YJ, Zhao YM, Chen J, Zhang XL. Carbon Nitride-based Sirna vectors with self‐produced O2 effects for Targeting Combination Therapy of Liver Fibrosis via HIF‐1α‐mediated TGF‐β1/Smad pathway. Adv Healthc Mater. 2023:2301485. 10.1002/adhm.202301485 37463681

[38] Ellison GD, Bresler DE (1974). Tests of emotional behavior in rats following depletion of norepinephrine, of serotonin, or of both. Psychopharmacologia.

[39] Crawley JN (1999). Behavioral phenotyping of transgenic and knockout mice: experimental design and evaluation of general health, sensory functions, motor abilities, and specific behavioral tests. Brain Res.

[40] Steru L, Chermat R, Thierry B, Simon P (1985). The tail suspension test: a new method for screening antidepressants in mice. Psychopharmacology.

[41] Petit-Demouliere B, Chenu F, Bourin M (2005). Forced swimming test in mice: a review of antidepressant activity. Psychopharmacology.

[42] Haapakoski R, Mathieu J, Ebmeier KP, Alenius H, Kivimäki M (2015). Cumulative meta-analysis of interleukins 6 and 1β, tumour necrosis factor α and C-reactive protein in patients with major depressive disorder. Brain Behav Immun.

[43] Howren MB, Lamkin DM, Suls J (2009). Associations of depression with C-reactive protein, IL-1, and IL-6: a meta-analysis. Psychosom Med.

[44] Perrin AJ, Horowitz MA, Roelofs J, Zunszain PA, Pariante CM (2019). Glucocorticoid resistance: is it a requisite for increased cytokine production in depression? A systematic review and meta-analysis. Front Psychiatry.

[45] Valkanova V, Ebmeier KP, Allan CL (2013). CRP, IL-6 and depression: a systematic review and meta-analysis of longitudinal studies. J Affect Disord.

[46] Zunszain PA, Anacker C, Cattaneo A, Carvalho LA, Pariante CM (2011). Glucocorticoids, cytokines and brain abnormalities in depression. Prog Neuropsychopharmacol Biol Psychiatry.

[47] Zito S, Nosari G, Pigoni A, Moltrasio C, Delvecchio G. Association between testosterone levels and mood disorders: a minireview. J Affect Disord. 2023. 10.1016/j.jad.2023.02.108 36841309

[48] Xu D-H, Du J-K, Liu S-Y, Zhang H, Yang L, Zhu X-Y, Liu Y-J (2023). Upregulation of KLK8 contributes to CUMS-induced hippocampal neuronal apoptosis by cleaving NCAM1. Cell Death Dis.

[49] Chen Y-B, Qiao T, Wang Y-Q, Cui Y-L, Wang Q-S (2022). Hydrogen bond-enhanced nanogel delivery system for potential intranasal therapy of Parkinson’s Disease. Mater Des.

[50] Zhou XH, He XL, Shi K, Yuan LP, Yang Y, Liu QY, Ming Y, Yi C, Qian ZY (2020). Injectable Thermosensitive Hydrogel Containing Erlotinib-Loaded Hollow Mesoporous silica nanoparticles as a localized drug delivery system for NSCLC Therapy. Adv Sci.

