# Supplementary material for: Enhanced antidepressant effects of BDNF-quercetin alginate nanogels for depression therapy
Source: J Nanobiotechnology. 2023 Oct 18;21:379. doi: 10.1186/s12951-023-02150-4 (PMC10583373; doi:10.1186/s12951-023-02150-4)
Supplement: Supplementary file 1 — Supplementary Material 1 [file 12951_2023_2150_MOESM1_ESM.docx]

**Supporting materials**

**Enhanced antidepressant effects of BDNF-Quercetin alginate nanogels for depression therapy**

Dong Xu ^1, 2, #^, Li-Na Gao ^1, 4#^, Xu-Jiao Song ^1, 2^, Qin-Wei Dong ^1, 2^, Yi-Bing Chen ^1, 2^, Yuan-Lu Cui ^1, 2,^ *, Qiang-Song Wang ^3,^ *

*^1^ State Key Laboratory of Component-based Chinese Medicine, Research Center of Traditional Chinese Medicine, Tianjin University of Traditional Chinese Medicine, Tianjin 301617, China*

*^2^ Haihe Laboratory of Modern Chinese Medicine, Tianjin 301617, China*

*^3^* *State Key Laboratory of Advanced Medical Materials and Devices, Engineering Research Center of Pulmonary and Critical Care Medicine Technology and Device (Ministry of Education), Tianjin Key Laboratory of Biomedical Materials, Institute of Biomedical Engineering, Chinese Academy of Medical Science & Peking Union Medical College, Tianjin 300192, China*

*^4^ Shandong Collaborative Innovation Center for Diagnosis, Treatment and Behavioral Interventions of Mental Disorders, Institute of Mental Health, College of Pharmacy, Jining Medical University, Jining 272067, Shandong, China*

^#^Indicates equal contribution

*Corresponding Author

Prof. Yuan-Lu Cui

No. 10 Poyanghu Road, West district of Tuanbo new town, Jinghai District, Tianjin, 301617, P R China

Fax: +86-22-59596229

E-mail: ylcui@tjutcm.edu.cn

Prof. Qiangsong Wang

No. 236, Baidi Road, Nankai District, Tianjin, 300192, P R China

Tel/Fax.: +86-22-87890919

E-mail: wangqs@bme.cams.cn

**S1 Chemicals and Materials:**

Quercetin (purity 98%) was purchased from Sangon Biotech, Co., Ltd. (Shanghai, China). Fluoxetine hydrochloride was from Eli Lilly and Company (Suzhou, Jiangsu, China). Brain-derived neurotrophic factor (BDNF) was bought from PeproTech, Inc. (Rocky Hill, NJ, USA). Lipopolysaccharide (LPS), sodium alginate (SA), the rhodamin B isothiocyanate (RBITC), Span 80, Tween 80, Poloxamer 407 (P 407), and Poloxamer 188 (P 188) were provided from Sigma-Aldrich Co. (St. Louis, MO, USA). UNIQ-10 column Trizol total RNA extraction kit was bought from Sangon Biotech, Co., Ltd. (Shanghai, China). Prostaglandin E_2_ Parameter Assay Kit was supplied by R&D Systems (Minneapolis, MN, USA). ACTH CLIA, corticosterone, CRH, testosterone, IL-6 CLIA ELISA Assay Kit was bought from Youersheng Science and Technology Co. Ltd. (Wuhan, Hubei, China). The colorimetric TUNEL apoptosis assay kit was from Beyotime Biotechnology Incorporation (Beijing, China). AntiMKI67 rabbit polyclonal antibody, FITC-conjugated IgG, was bought from Sangon Biotech, Co., Ltd. (Shanghai, China). Anti-BDNF rabbit polyclonal antibody, anti-Motor (Phospho-Ser2481, p-mTOR) rabbit polyclonal antibody, and anti-GAPDH antibody were bought from Sangon Biotech, Co., Ltd. (Shanghai, China). GRIN2B and GRIA3 polyclonal antibodies were purchased from eBioscience (San Diego, CA, USA). Anti-TrkB polyclonal antibody was from Abgent (San Diego, CA). Anti-rabbit IgG (whole molecule)-peroxidase produced in goats was provided by Sigma-Aldrich Co. (St. Louis, MO, USA). The RAW 264.7 cells were maintained in a 37 °C incubator with a humidified 5% CO_2_ atmosphere. All other commercially available chemicals in this article were analytical grade and used without further purification.

**S2 Animals:**

Male SD rats (180-220 g) and male ICR mice (18-22 g) were obtained from the Vital River Laboratory Animal Technology Co., Ltd. (Beijing, China). The experimental animals were fed at room temperature (24 ± 1 °C) and relative humidity (56 ± 5%). All animal procedures were performed by the Guidelines for Care and Use of Laboratory Animals of Tianjin University of Traditional Chinese Medicine.

**S3 Preparation and characterization of BDNF-Quercetin nanogels:**

Paraffin oil (150 mL) containing 1.05 mL of Span 80 and 0.45 mL of Tween 80 was mixed for 30 min under stirring of 500 rpm. Next, 45 mL sodium alginate (SA, 0.5%, g/mL) solution containing BDNF (250 μg) was dropped into the above-dispersed phase, continuously stirring for 1 hour at 1000 rpm. To better form BDNF-Quercetin nanogels, ZnCl_2_ solution of different concentrations was dropped into the mixed solution by two steps, namely 60% ethanol (7 mL) containing 0.02% of ZnCl_2_ and 0.3% of quercetin and 60% ethanol (7 mL) containing 0.18% of ZnCl_2_. Finally, BDNF-Quercetin nanogels in the lower layer were subjected to centrifugation and then lyophilized for 24 h. Quercetin nanogels were also obtained as a protein carrier for characterization. Blank nanogels, without BDNF and quercetin, were also collected as controls.

The particle size distribution of quercetin nanogels was determined by phase-analysis light scattering (PALS) using a Zetasizer Nano-ZS (NanoZS, Malvern Instruments, UK). The morphology and shape of quercetin nanogels were observed using a transmission electron microscope (TEM) (H-7650, Hitachi, Tokyo, Japan). The construction of quercetin nanogels was characterized by differential scanning calorimetry (Jade DSC, Perkin-Elmer Corp, Wilton, USA), Rigaku D/max-2500 X-ray diffractometer (Rigaku, Japan) with Cu Kα radiation, Confocal Raman microscopy (Renishaw, UK), and Thermo Nicolet iS10 Fourier Transform Infrared (FT-IR) spectrometer (Thermo Scientific, MA, USA).

The quercetin loading capacity of nanogels was detected by a 2695 high-performance liquid chromatography (HPLC) system (Waters, NY, USA) with a Kromasil 100-5 C_18_ column (4.6 mm × 250mm × 5 μm, Sweden). The mobile phase solvent consisting of methanol and 0.1% phosphoric acid solution (85/15, v/v) was pumped at a flow rate of 1.0 mL min^−1^. Detection was performed at a wavelength of 280 nm. The BDNF loading capacity of nanogel was assessed using the BDNF Emax® Immunoassay System, which was from Promega Corporation (Madison, Wisconsin, USA) according to the manufacturer's instructions. Finally, quercetin and BDNF loading efficiency were calculated according to the following equations:

$$Drug loading efficiency \left( \% \right)= \frac{Amount of drug in nanogels}{Amount of nanogels}\times100\%$$

**S4 Antioxidant activities of quercetin nanogels**

In brief, albumin solution, albumin solution combined with quercetin were added into H_2_O_2_ and vortexed, respectively. Next, Coomassie brilliant blue was mixed with the above solution and vortexed. The final sample was added to a 96-well plate, and its absorbance was measured at 595 nm using a microplate reader (Infinite M200, Tecan Co.). The DPPH scavenging rate of quercetin nanogel was determined by the previous methods. The sample (quercetin solution and quercetin nanogels) and DPPH were dissolved in methanol as Ai. The methanol was used to replace DPPH as A0. The mixture of methanol and DPPH was as Amax. The mixture was added to the 96-well plate, and three parallel operations were done. The mixture was reacted in the dark at 25 °C for 15 min. OD values of the testing samples were detected at 517 nm.

**S5 Cell biology evaluation and immune response**

RAW 264.7 cells were treated with blank nanogels (100, 200, 400 μg/mL), quercetin (1, 2, 4 μg/mL), LPS (0.2 μg/mL), quercetin nanogels (1, 2, 4 μg/mL) for 24 h or 48h. The cell proliferation was detected using the BrdU ELISA kit of Roche Molecular Biochemicals (Indianapolis, IN, USA). Calcein-AM fluorescence assay was used to observe cell viability under the inverted fluorescence microscope (Nikon ECLIPSE, Ti-E, Japan). Nitric oxide contents were measured by laser scanning confocal microscopy (Carl Zeiss Microimaging Inc., Germany) using 4-amino-5-methylamino-2′, 7′-dichlorofluorescein (DAF-FM). Nitric oxide (NO) secretion was detected using a Griess reaction. According to the manufacturer's instructions, IL-6, TNF-α, and PGE2 protein levels were detected using ELISA Assay bought from eBioscience (San Diego, CA, USA). The protein expression of COX-2 was performed using a cell-based ELISA assay. RAW 264.7 cells were collected, and its total RNA was isolated using a Sangon UNIQ-10 column Trizol total RNA extraction kit according to the manufacturer's instructions. The real-time RT-PCR primers were demonstrated in **Table S2**, and then the reactions were performed in a Bio-Rad C1000 (Bio-Rad, Pleasanton, CA, USA). The folds increase or decrease was calculated relative to blank control after normalized to a housekeeping gene using the 2^-ΔΔCT^ method.

**S6 Pharmacokinetic study**

At each time point (5, 10, 15, 30, 60, 120, 240, 360, 480, 720, and 1440 min), the hippocampi were collected from rat brains after anesthesia and transferred to a centrifuge tube. Rat hippocampal tissue (m/v=1:5) was added tissue lysate (methanol: acetonitrile: acetone, 40:40:20, v/v/v) and 5 μL puerarin (5 μg/mL) to break up the tissue. After standing on ice for 10 min, the tissue solution was centrifuged at 16,000 × g for 10 min, and 250 μL of the supernatant was taken to dry up with nitrogen and analyzed by LC-MS/MS. The HPLC system consisting of a mobile phase (methanol: 0.1% phosphoric acid solution = 85/15 v/v) was used for pharmacokinetic analysis. Relative bioavailability was calculated according to the following equation.

$$Relative biovailability=\frac{{AUC}_{nano}}{{AUC}_{sol}}\times\frac{{Dose}_{sol}}{{Dose}_{nano}}\times100\%$$

AUC_nano_ and AUC_sol_ are the areas under the concentration-time curve from time 0 to 24 h after intranasal or intragastric administration of quercetin nanogels and quercetin solution (calculated as the weight of quercetin), respectively. Dose_nano_ and Dose_sol_ are the dosage of quercetin nanogels and quercetin solution, respectively.

**S7 Open field test (OFT) and forced swim test (FST), tail suspension test (TST), and reserpine-induced depression model**

Antidepressant activities of the mice were evaluated by open field test (OFT), forced swim test (FST), and tail suspension test (TST). The spontaneous activity of the mice was assessed using OFT. In brief, the mice were randomly divided into 10 groups (12 mice/group) which include control (0.9% normal saline, intranasal administration), fluoxetine (14 mg/kg, intragastric administration), quercetin (28 mg/kg, intragastric administration), quercetin nanogels in thermosensitive gel (0.05, 0.1, 0.2, 0.4, 0.8 mg/kg calculated by the weight of quercetin, intranasal administration), and BDNF-Quercetin nanogels in thermosensitive gel containing quercetin (0.1, 0.2 mg/kg) and BDNF (3, 6 μg/kg) via intranasal administration. The mice were individually placed in a black box (50 × 50× 40 cm) one hour after administration. The total distance and number of rearing of the mice during the last 4 min of the 6 min test were recorded using behavioral equipment (Multifunctional 256060 Series, TSE Systems, Germany). The behavioral despair of the mice at the end of the experimental period was elevated by FST and TST according to the group of OFT. One hour after administration, for FST, the mice were forced to swim in polymethyl methacrylate cylinders (20 cm height, 10 cm diameter) containing 15 cm water depth at 25 ± 1 °C for 6 min. For the TST, the mice were individually suspended 15 cm above the floor by adhesive tape placed approximately 1 cm from the tip of the tail for 6 min. The last 4 min of the 6 min in FST and TST test was used to analyze the immobility duration of the mice via behavioral equipment (Multifunctional 256060 Series, TSE Systems, Germany).

A reserpine-induced depression model of monoamine neurotransmitter depletion was used to evaluate the antidepressant effects of the preparations. The rats were randomly divided into 7 groups, namely control (0.9% normal saline, intranasal administration), model (0.9% normal saline, intranasal administration), fluoxetine (7 mg/kg, intraperitoneal injection), quercetin (20 mg/kg, intragastric administration), quercetin nanogels in thermosensitive gel (0.075, 0.15, and 0.3 mg/kg calculated by the weight of quercetin, intranasal administration). Expect for control, the rats were intraperitoneally injected into reserpine 0.5 h after administration. The ptosis score of rats was observed 1 h after being injected with reserpine. Next, the rats were sacrificed, and then the hippocampi and striatum were collected. The monoamine neurotransmitters, including 5-HT, NE, and DA in the hippocampi and striatum, were detected using an electrochemical detector (Waters, NY, USA) with a Capcell pakC_18_ Column (3.0 mmL.D x 75mm, 3μM).

**S8 The procedure of RNA sequencing**

According to the manufacturer's instructions, total RNA was extracted from rat hippocampi (4 replicates/group) with TRIzol (Invitrogen, USA). Using the NEBNext Ultra RNA Library Prep Kit to prepare the sequencing library, the index code was added to the attribute sequence of each sample. The library was prepared and sequenced on the HiSeq 2500 platform (Illumina, USA). The original data is processed by in-house Perl scripts, and the clean data is obtained by deleting reads containing adapters, reads containing poly-N, and low-quality data read from the original data. The sequencing quality and trim reads were examined using the FASTQ and FASTX toolbox. Sequences were aligned using TopHat2 to Rattus norvegicus Ensembl Rnor 5.0 assemble. The reads that appear in multiple genes are removed from the analysis. In at least 80% of the samples, the threshold for screening low-level expressed genes is set to >5 reads.

**S9 Determination of hippocampal tissues in metabolite levels**

Every group includes eight replicate samples. The metabolomic analysis was performed on an LC-MS/MS system coupled with a Shimadzu LC-20AD Qtrap 5500 tandem mass spectrum (SCIEX, United States). Ten microliters of the extracts were injected into an apHera NH2 HPLC column (150 × 2 mm, 4 μm, Supelco, United States) held at 25°C for chromatographic separation. In summary, 625 metabolites were targeted to predict the main metabolomic pathway. Metabolomics data were processed by the metaboanalyst platform (<http://www.metaboanalyst.ca/>).

**S10 Western blotting detection**

Total protein in rat hippocampal tissues was extracted by a mammalian protein extraction kit following the manufacturer's instruction (Sangon Biotech, Shanghai, China). The BCA method (Thermo Fisher, IL, USA) was used to quantify protein. An amount of 40 mg protein was resolved on 10% SDS-PAGE gels and transferred to PVDF membranes. The PVDF membranes were incubated with blocking solution (5% non-fat milk) in Tris-buffered saline containing 0.1% Tween 20 (TBST) for 1.5 h with shaking at room temperature and then incubated overnight with anti-GRIA3, anti-GRIN2B, anti-BDNF, anti-TrkB, anti-GSK3β, anti-GAPDH antibody at 4 °C. After being washed with TBST for five times, the membrane was incubated with secondary antibodies conjugated to horseradish peroxidase for 1 h at room temperature. The PVDF membranes were washed five times with TBST again and examined using an enhanced chemiluminescence detection kit (EZ-ECL, Biological Industries, and Israel) on the films, exposing to Kodak BioMax Light films. The PVDF membranes were scanned and quantified by Image J (version 1.53q) and then normalized to the corresponding GAPDH intensity as control.

**Table S1 Fitting analysis of quercetin and BDNF released from** **BDNF-Quercetin nanogels in the thermosensitive gel.**

| **Drugs** | **Model** | **Equation** | **R^2^** |
| --- | --- | --- | --- |
| Quercetin | Zero-order | Q = 3.58t+22.64 | 0.6435 |
|  | First-order | Q = 83.84(1-e^-0.27t^) | 0.9957 |
|  | Higuchi | Q = 20.56t^1/2^ +3.85 | 0.8962 |
|  | Ritger-Peppas | Q = 27.01t^0.41^ | 0.9203 |
| BDNF | Zero-order | Q = 3.89t+41.70 | 0.4020 |
|  | First-order | Q=103.30(1-e^-0.53t^) | 0.9915 |
|  | Higuchi | Q = 24.60t^1/2^ +17.33 | 0.7148 |
|  | Ritger-Peppas | Q = 47.28t^0.31^ | 0.8157 |

Where t is release time. Q is the cumulative release amount from BDNF-Quercetin nanogels in the release medium.

**Table S2. The real-time RT-PCR oligonucleotide primers of inflammatory gene.**

| Gene | Primer | Sequence (5’-3’) | PCR product (bp) |
| --- | --- | --- | --- |
| β-actin | Forward | TGTTACCAACTGGGACGACA | 165 |
| (NM_007393.3) | Reverse | GGGGTGTTGAAGGTCTCAAA |  |
| NOS II | Forward | CACCTTGGAGTTCACCCAGT | 170 |
| (NM_010927.3) | Reverse | ACCACTCGTACTTGGGATGC |  |
| COX-2 | Forward | TGAGTACCGCAAACGCTTCTC | 151 |
| (NM_011198.3) | Reverse | TGGACGAGGTTTTTCCACCAG |  |
| IL-6 | Forward | TCCAGTTGCCTTCTTGGGAC | 140 |
| (NM_031168.1) | Reverse | GTGTAATTAAGCCTCCGACTTG |  |
| TNF-α | Forward | TAGCCAGGAGGGAGAACAGA | 127 |
| (NM_013693.2) | Reverse | TTTTCTGGAGGGAGATGTGG |  |

**Table S3. The real-time RT-PCR oligonucleotide primers of apoptosis gene.**

| Gene | Primer | Sequence (5’-3’) | PCR product (bp) |
| --- | --- | --- | --- |
| GAPDH | Forward | AGACAGCCGCATCTTCTTGT | 142 |
| (NM_017008.4) | Reverse | TGATGGCAACAATGTCCACT |  |
| Bcl-2 | Forward | AAGCTGTCACAGAGGGGCTA | 97 |
| (NM_016993.1) | Reverse | CAGGCTGGAAGGAGAAGATG |  |
| Bax | Forward | CGAGCTGATCAGAACCATCA | 191 |
| (NM_017059.2) | Reverse | CTCAGCCCATCTTCTTCCAG |  |
| Bcl-xL | Forward | GCTGGTGGTTGACTTTC | 148 |
| (NM_001033671.1) | Reverse | GGATGGGTTGCCATTGA |  |

**Table S4. GO enrichment analysis related to antioxidant activity.**

| Number | Description | Term_type |
| --- | --- | --- |
| 1 | oxidation-reduction process | biological_process |
| 2 | energy derivation by oxidation of organic compounds | biological_process |
| 3 | oxidative phosphorylation | biological_process |
| 4 | antioxidant activity | molecular_function |
| 5 | cytochrome-c oxidase activity | molecular_function |
| 6 | peroxidase activity | molecular_function |
| 7 | sarcosine oxidase activity | molecular_function |
| 8 | heme-copper terminal oxidase activity | molecular_function |
| 9 | energy derivation by oxidation of reduced inorganic compounds | biological_process |
| 10 | alternative oxidase activity | molecular_function |
| 11 | pyridoxamine-phosphate oxidase activity | molecular_function |
| 12 | glutathione peroxidase activity | molecular_function |
| 13 | thiol oxidase activity | molecular_function |
| 14 | response to oxidative stress | biological_process |
| 15 | cellular response to oxidative stress | biological_process |


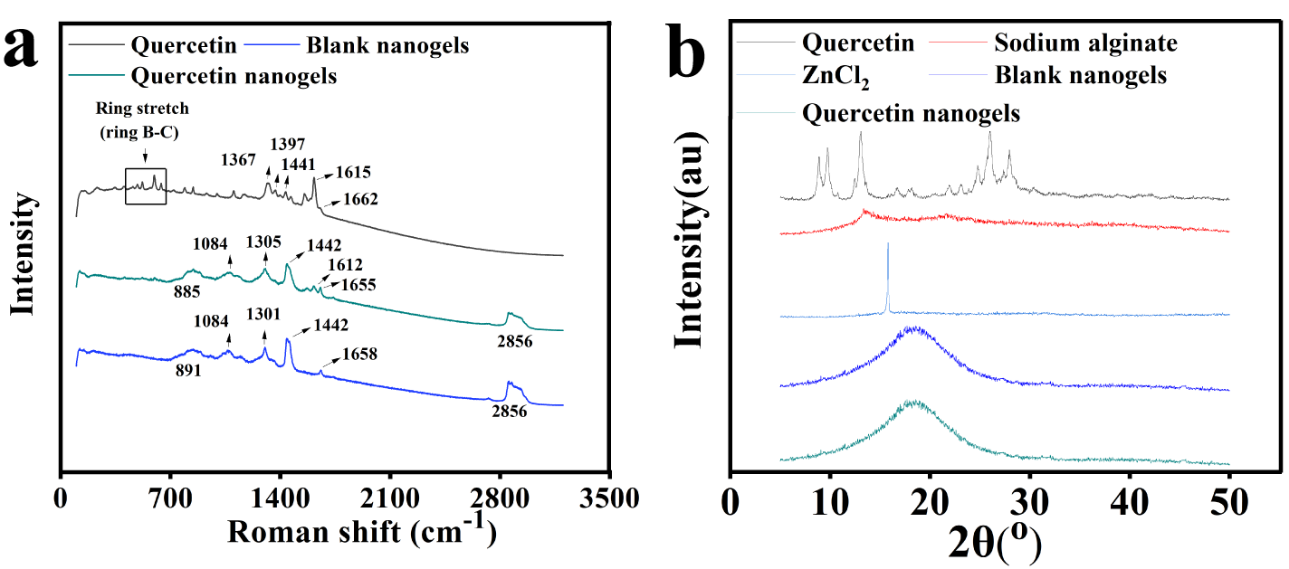


**Figure S1 The characterization of quercetin nanogels.** a. Confocal micro-Raman spectroscopy. b. X-ray diffraction analysis.


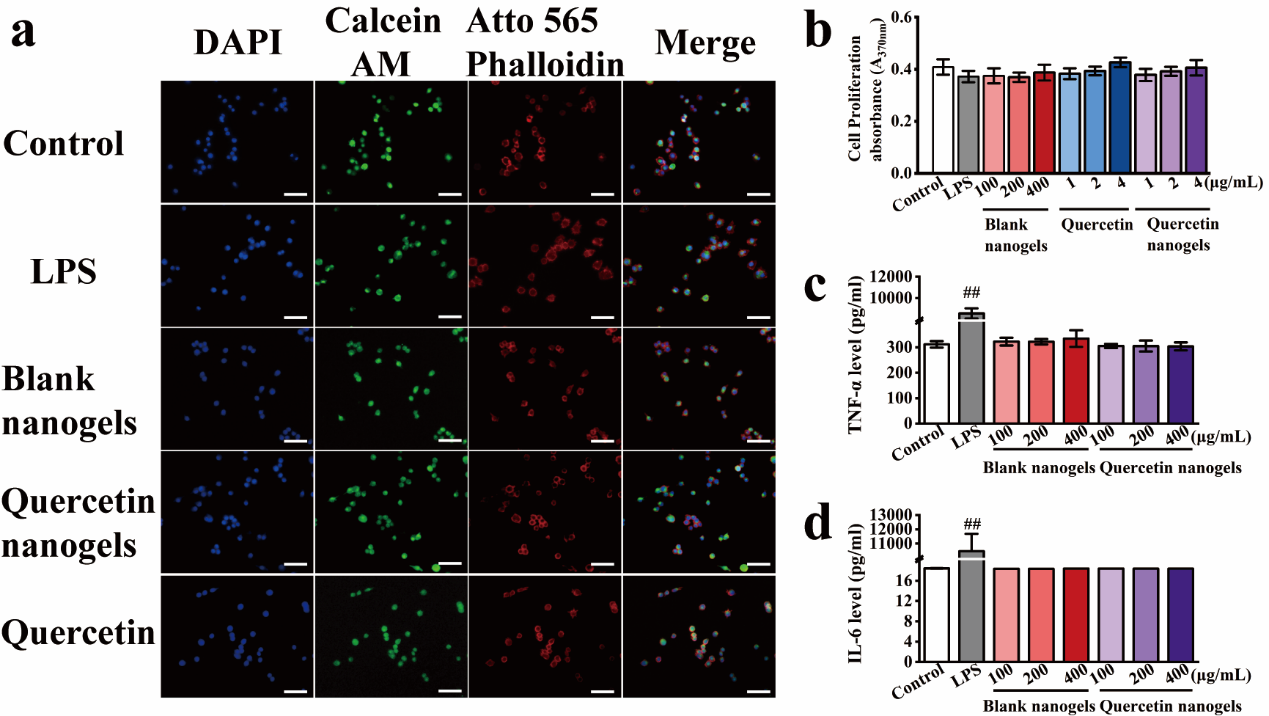


**Figure S2 Cell biology evaluation and immune response of quercetin nanogels.** (a) Cell viability. (b) Cell proliferation. (c) TNF-α level. (d) IL-6 level. *^##^p* < 0.01 compared with the control group.


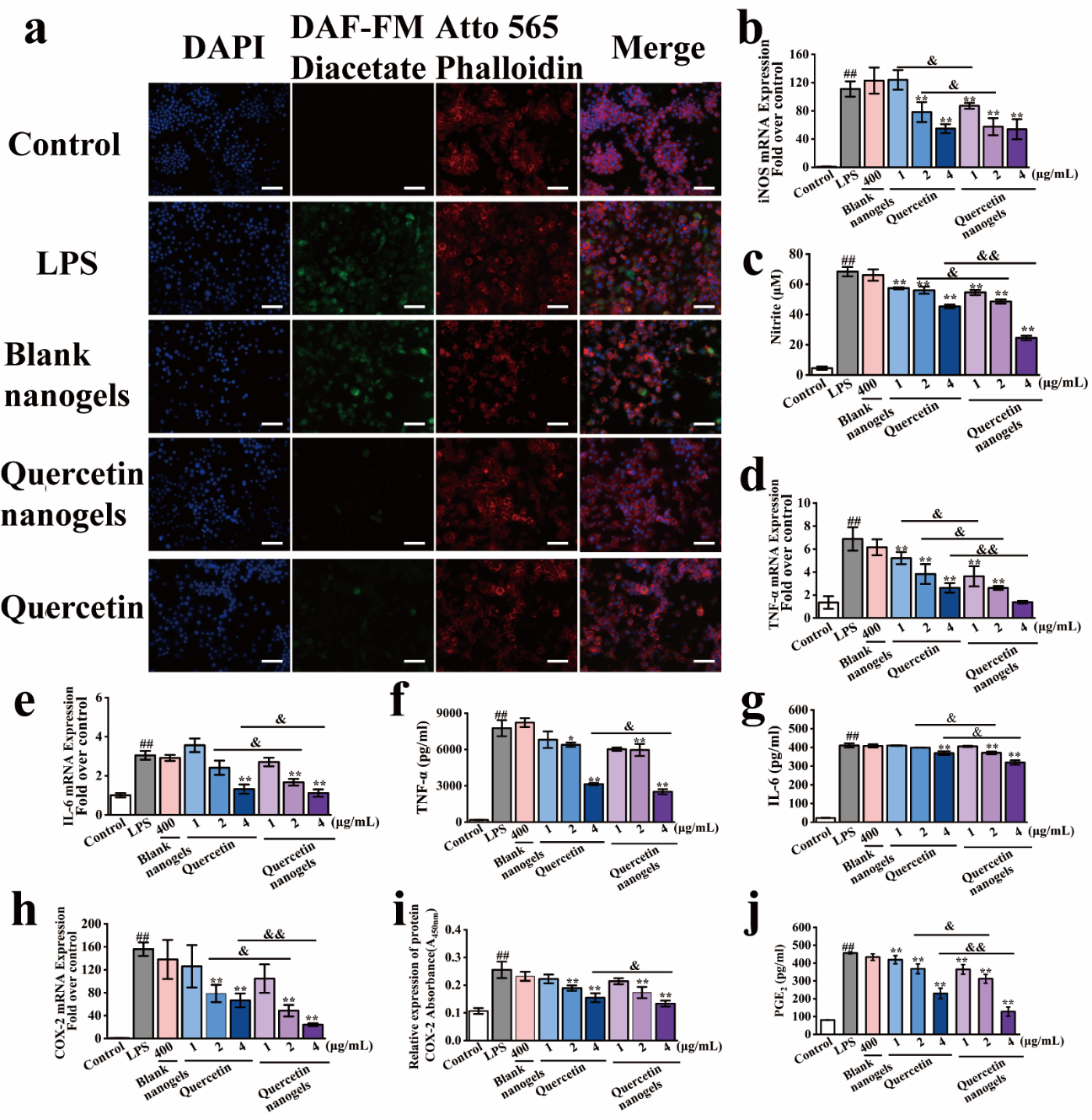


**Figure S3 Anti-inflammatory activities of quercetin nanogels in LPS-induced RAW264.7 cells.** (a) Fluorescence micrograph of NO staining in RAW 264.7 cells. (b) iNOS mRNA expression. (c) Secretion of nitrite. (d) TNF-α mRNA expression. (e) IL-6 mRNA expression. (f) TNF-α protein expression. (g) IL-6 protein expression. (h) COX-2 mRNA expression. (i) COX-2 protein expression. (j) PGE_2_ protein expression. *^##^p* < 0.01 compared with the control group. ^*^*p* < 0.05, ^**^*p* < 0.01 compared with LPS group. ^&^*p* < 0.05, ^&&^*p* < 0.01 compared with quercetin group.


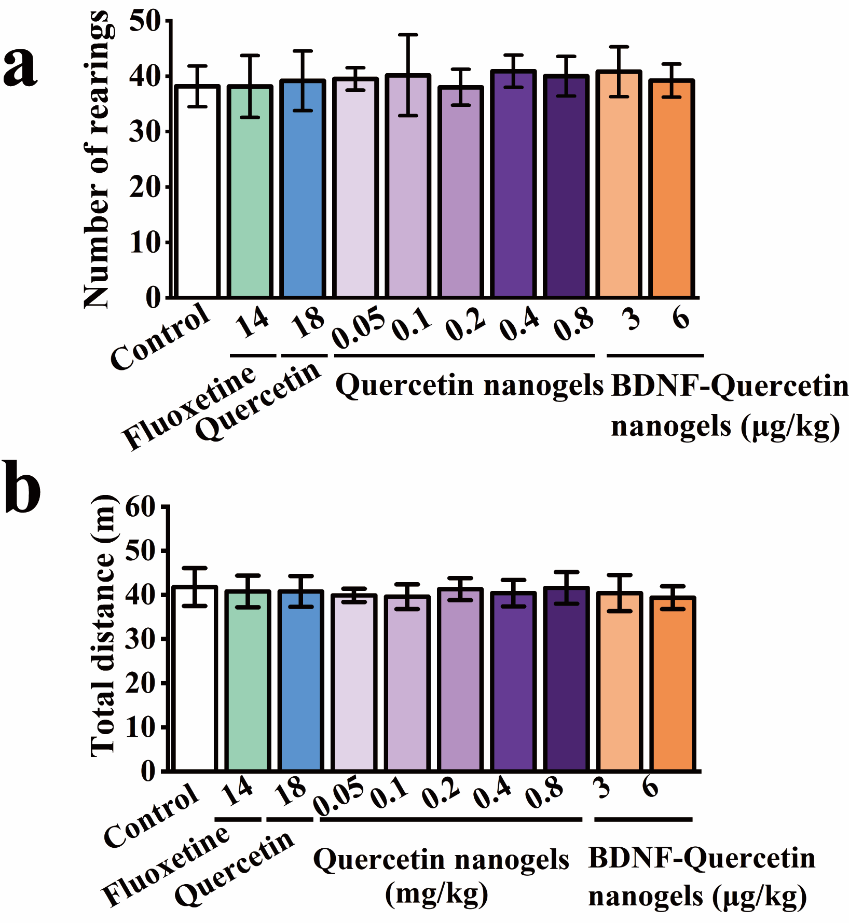


**Figure S4 Open field test of BDNF-Quercetin nanogels on the mice.** (a) Number of rearings. (b) Total distance.


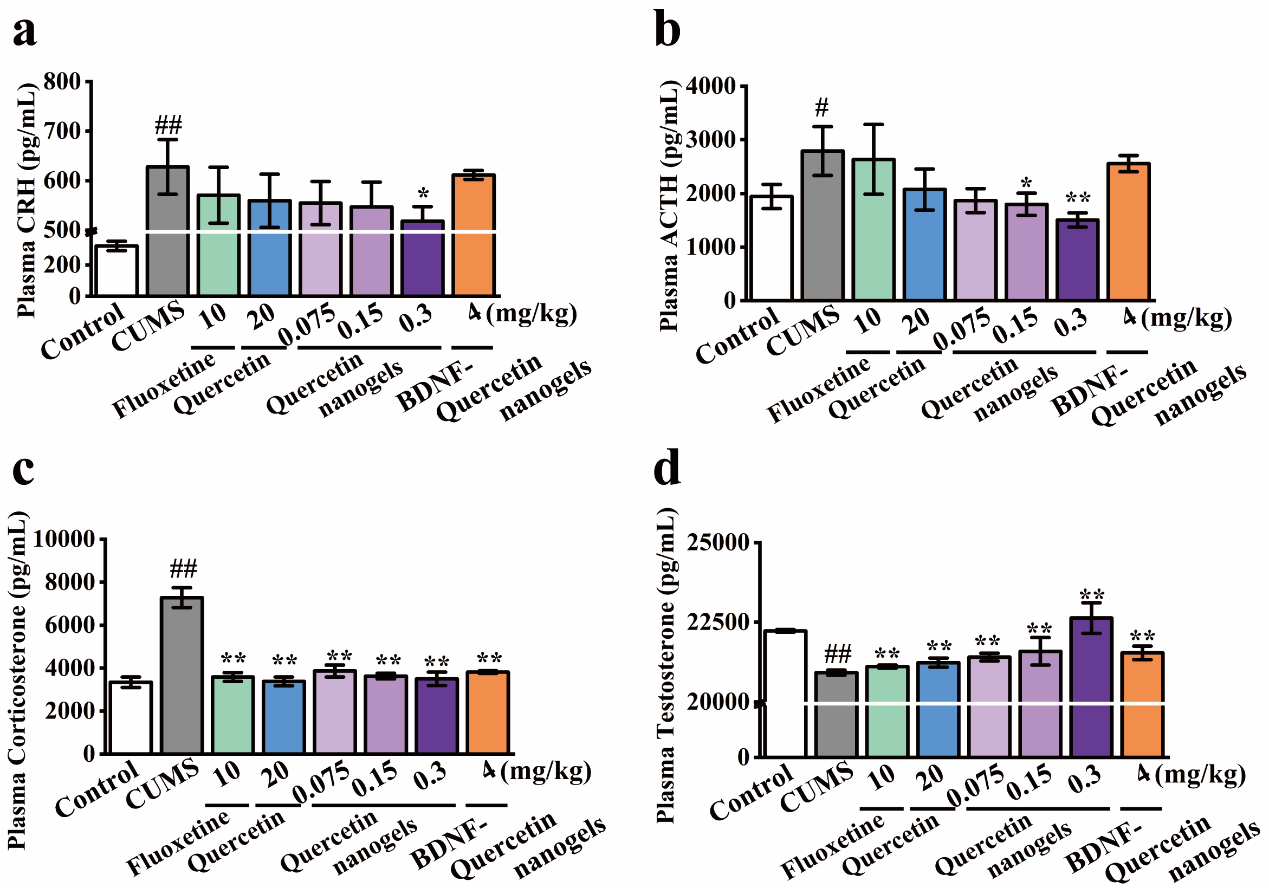


**Figure S5 Regulation of BDNF-Quercetin nanogels on the hypothalamic-pituitary-adrenal (HPA) axis**. (a) Plasma CRH (pg/mL). (b) Plasma ACTH (pg/mL). (c) Plasma corticosterone (pg/mL). (d) Plasma testosterone (pg/mL).


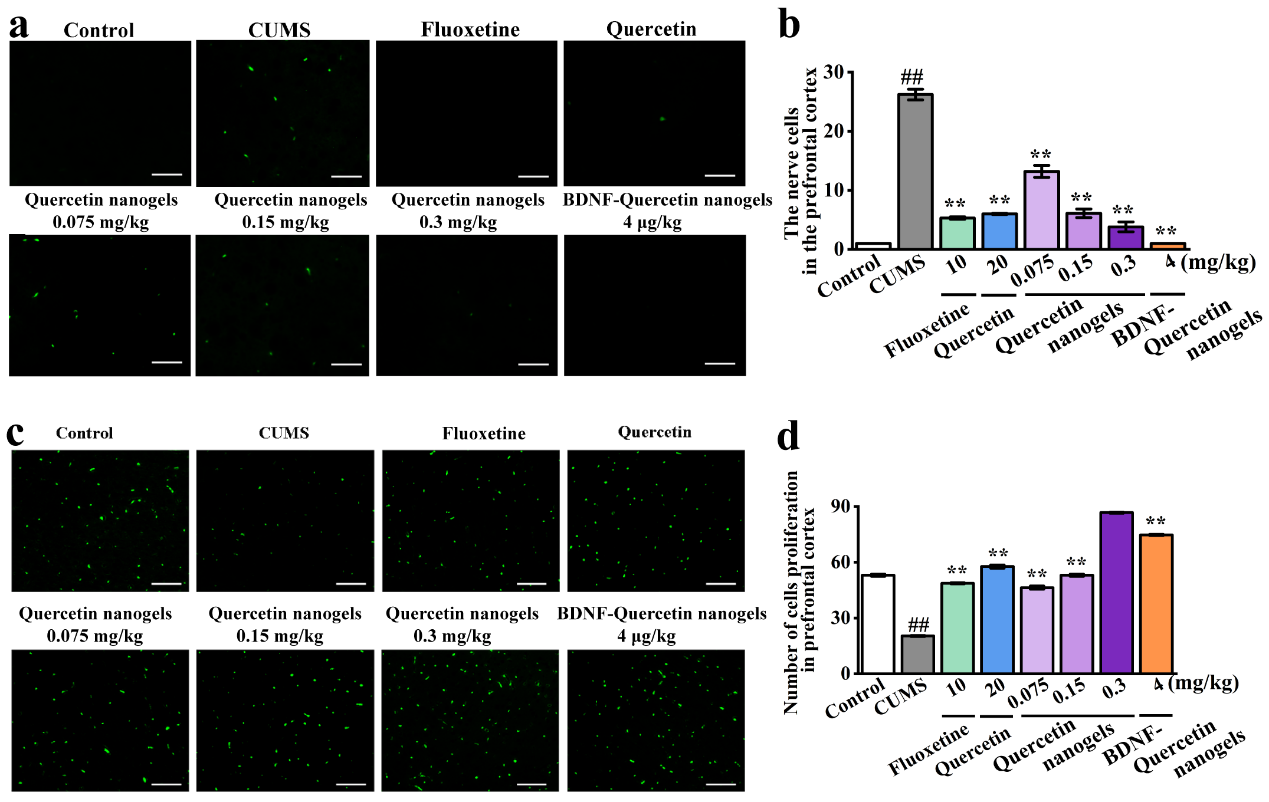


**Figure S6 CUMS-induced cell apoptosis and proliferation in the** **prefrontal cortex of rats.** (a) TUNEL staining. (b) The nerve cells by TUNEL staining. (c) Ki67 staining. (d) Number of cell proliferation by Ki67 staining. ^##^*p* < 0.01 compared with the control group. ^*^*p* < 0.05, ^**^*p* < 0.01 compared with the CUMS group.


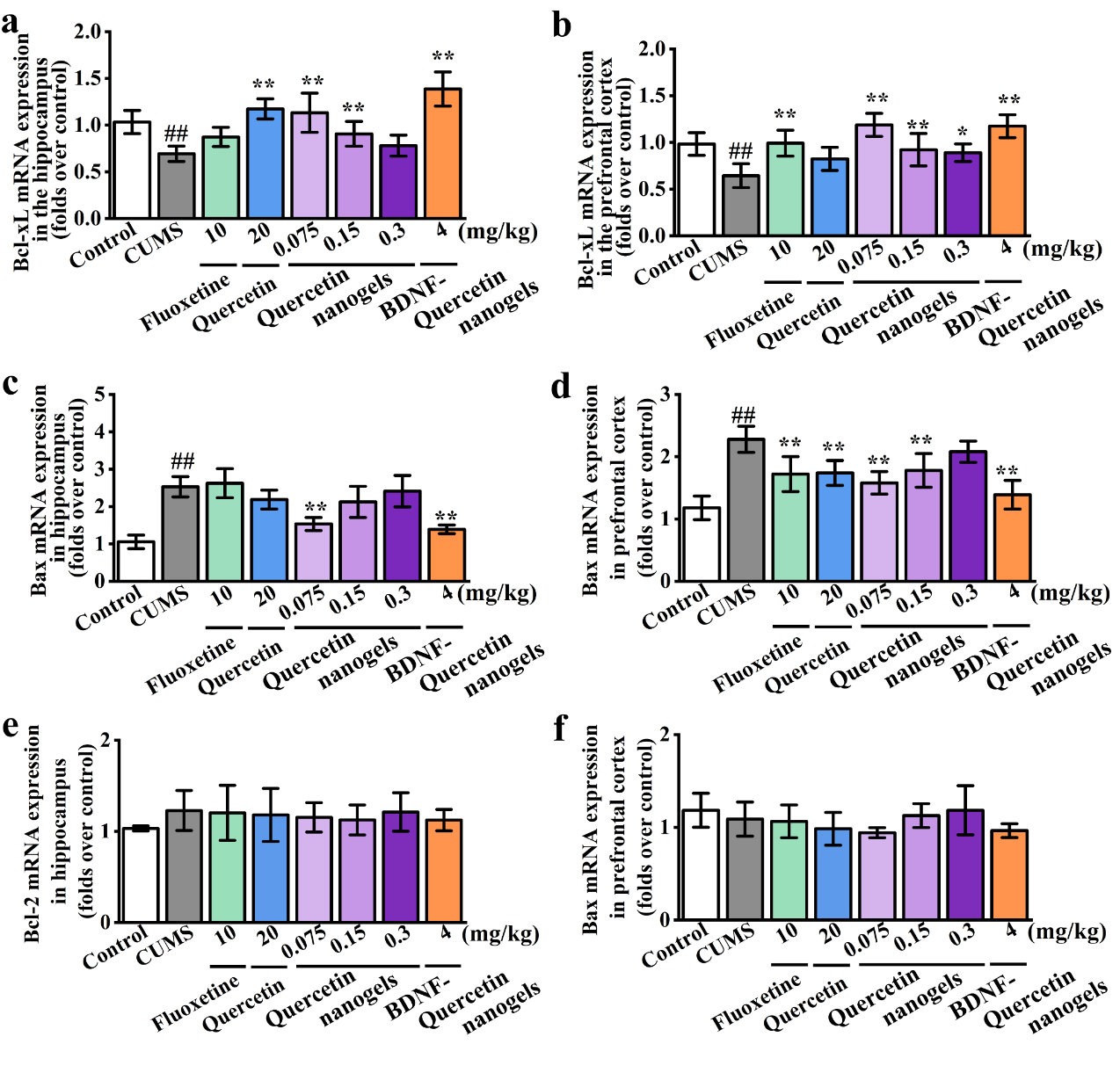


**Figure S7 Expression of apoptotic factors in rat hippocampal and prefrontal cortex.** (a) Bal-xL mRNA expression in the hippocampus of rats. (d) Bal-xL mRNA expression in the prefrontal cortex of rats. (c) Bax mRNA expression in the hippocampus of rats. (d) Bax mRNA expression in the prefrontal cortex of rats. (e) Bcl-2 mRNA expression in the hippocampus of rats. (f) Bcl-2 mRNA expression in the prefrontal cortex of rats. ^##^*p* < 0.01 compared with the control group. ^*^*p* < 0.05, ^**^*p* < 0.01 compared with the CUMS group.
